# Supplementary material for: Residue Folding Degree—Relationship to Secondary Structure Categories and Use as Collective Variable
Source: Int J Mol Sci. 2021 Dec 2;22(23):13042. doi: 10.3390/ijms222313042 (PMC8657879; doi:10.3390/ijms222313042)
Supplement: Supplementary file 1 [file ijms-22-13042-s001.zip › ijms-1460924-supplementary.pdf]

Supporting Information for: Residue Folding Degree, relationship to Secondary Structure Categories and use as Collective Variable.

Vladimir Sladek,[*,†] Ryuhei Harada,[*,‡] and Yasuteru Shigeta[*,‡]

†*Institute of Chemistry, Dubravska cesta 9, 84538 Bratislava, Slovakia*

‡*Center for Computational Sciences, University of Tsukuba, Tennodai 1-1-1, Tsukuba, Ibaraki 305-8577, Japan*

E-mail: sladek.vladimir@savba.sk; ryuhei@ccs.tsukuba.ac.jp; shigeta@ccs.tsukuba.ac.jp

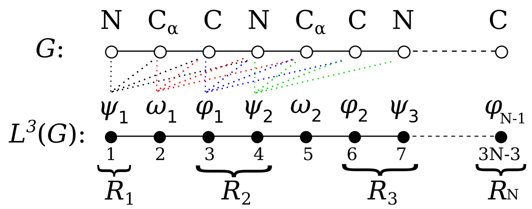

Figure S 1: Schematic representation of node correspondence between G, the path graph of the backbone atoms, and $L^3(G)$, the path graph of the backbone dihedral angles. We assume N residues in the peptide. The vertex contributions $C_S^{(i)}$ to $\langle C_S \rangle$ may be grouped to residue subgraph centrality terms $RC_S^{(k)}$, with k labelling the residues.[1] The contribution of the non-terminal residues to $\langle C_S \rangle$ is the subgraph centrality of two vertices (dihedrals), contrary to the terminal ones for which only one angle contributes. The contribution of ω, the dihedral angle of the peptide bond, is not assigned to residues. See also.[2]

Table S 1: Typical RC_S values for SS categories as defined by the DSSP 4, DSSP, and STRIDE algorithms. The mean values with (sample) standard deviations in parentheses are given for RC_S and the φ, ψ values. Count indicates how many residues were these numbers based on (fall in the category).

SS cat.	φ	ψ	RC_S	count
		DSSP 4		
H	-64.70 (11.84)	-39.56 (11.39)	7.273 (0.421)	412071
G	-66.05 (34.16)	-15.55 (29.33)	7.523 (0.840)	51822
I	-79.15 (25.74)	-41.63 (20.41)	6.516 (0.999)	7086
E	-110.89 (42.62)	122.38 (58.10)	2.730 (0.966)	282196
B	-96.98 (49.29)	122.90 (67.45)	2.976 (0.898)	15416
T	-39.33 (70.25)	6.23 (51.49)	6.923 (1.223)	151631
S	-69.26 (73.32)	44.16 (97.63)	4.850 (2.166)	113536
P	-72.33 (13.05)	144.79 (13.85)	3.660 (0.498)	24764
C	-82.97 (55.81)	97.00 (83.52)	3.835 (1.574)	218948
		DSSP		
H	-64.70 (11.84)	-39.56 (11.39)	7.273 (0.421)	412071
G	-66.05 (34.16)	-15.55 (29.33)	7.523 (0.840)	51822
I	-79.15 (25.74)	-41.63 (20.41)	6.516 (0.999)	7086
E	-110.89 (42.62)	122.38 (58.10)	2.730 (0.966)	282195
B	-96.98 (49.29)	122.90 (67.45)	2.976 (0.898)	15416
T	-39.33 (70.25)	6.23 (51.49)	6.923 (1.223)	151631
S	-69.26 (73.32)	44.16 (97.63)	4.850 (2.166)	113539
C	-81.89 (53.16)	101.86 (80.59)	3.817 (1.502)	243712
		STRIDE		
H	-65.99 (12.79)	-37.93 13.95)	7.257 (0.457)	430137
G	-65.93 (30.27)	-19.25 25.29)	7.556 (0.713)	54199
I	-69.98 (56.25)	-30.04 52.74)	6.100 (1.522)	225
E	-110.35 (42.80)	122.06 59.32)	2.742 (0.974)	290269
B	-98.91 (50.47)	119.07 75.72)	2.913 (0.879)	15318
T	-59.45 (67.24)	41.21 83.36)	5.560 (2.062)	271050
C	-72.44 (65.97)	86.14 88.48)	4.177 (1.828)	221302

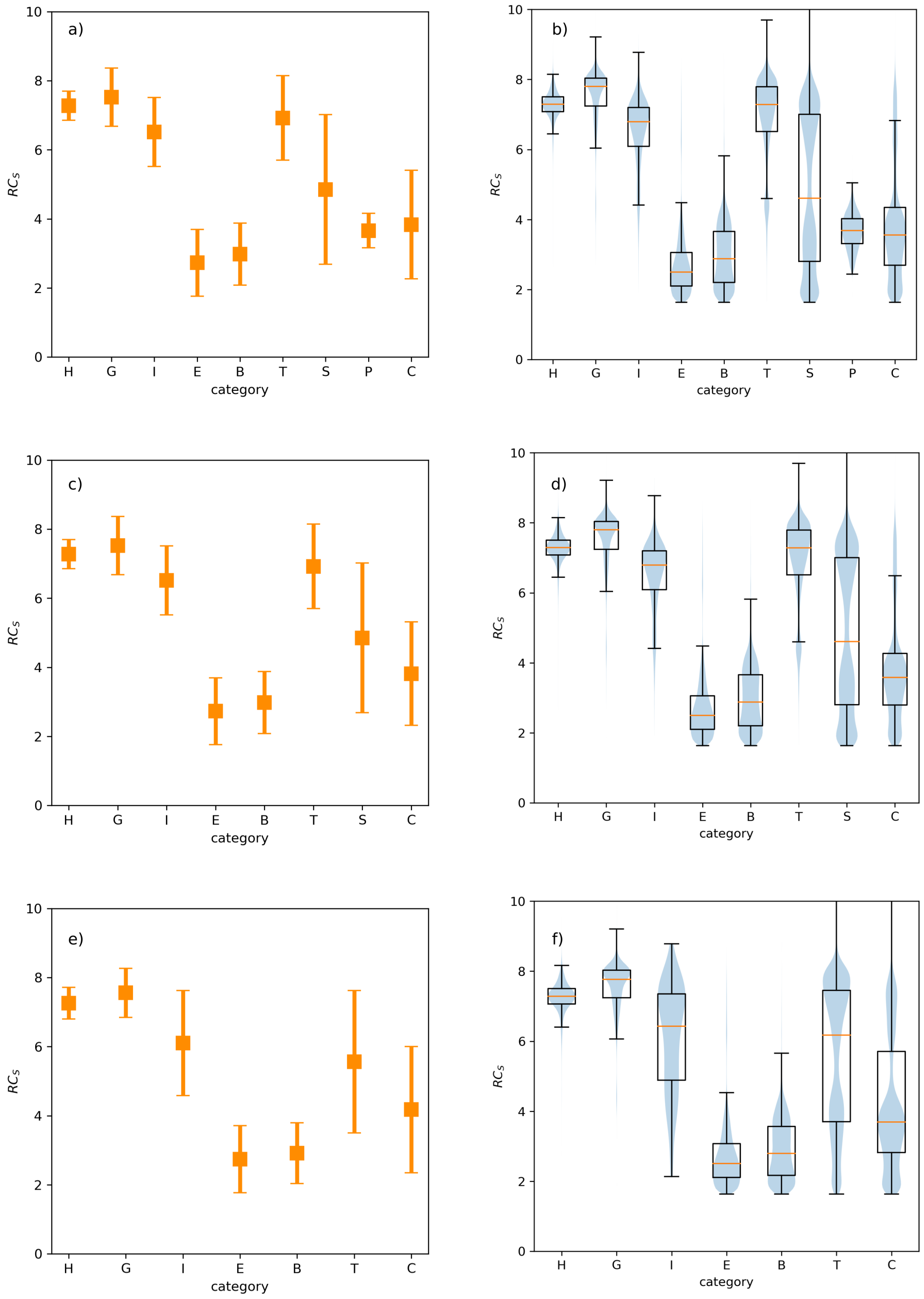

Figure S 2: Typical residue folding degree values for SS categories from DSSP 4 in a), b), DSSP in c), d), and STRIDE in e) f). The mean value and the standard deviations are shown in a) and the densities and box plots in b). The box plot also indicates the median Q_2 (orange line), the lower and upper quartiles Q_1, Q_3 (bottom and top of the box) and the whisker ends indicate statistical outliers at $Q_1 - 1.5(Q_3 - Q_1)$ and $Q_3 + 1.5(Q_3 - Q_1)$.

$$
\begin{array}{c}
 \\ H \\ G \\ I \\ E \\ B \\ T \\ S \\ P \\ C
\end{array}
\begin{array}{cccccccc}
H & G & I & E & B & T & S & C \\
412073 & 0 & 0 & 0 & 0 & 0 & 0 & 0 \\
0 & 51820 & 0 & 0 & 0 & 0 & 0 & 0 \\
0 & 0 & 7086 & 0 & 0 & 0 & 0 & 0 \\
0 & 0 & 0 & 282173 & 0 & 0 & 0 & 1 \\
0 & 0 & 0 & 0 & 15339 & 0 & 0 & 0 \\
0 & 0 & 0 & 0 & 0 & 151613 & 0 & 0 \\
0 & 0 & 0 & 0 & 0 & 0 & 113466 & 2 \\
0 & 0 & 0 & 0 & 0 & 0 & 0 & 24736 \\
0 & 0 & 0 & 0 & 0 & 0 & 5 & 228972
\end{array}
\tag{1}
$$

$$
\begin{array}{c}
 \\ H \\ G \\ I \\ E \\ B \\ T \\ S \\ P \\ C
\end{array}
\begin{array}{ccccccc}
H & G & I & E & B & T & C \\
398989 & 6772 & 2 & 6 & 10 & 4989 & 1305 \\
972 & 42995 & 2 & 52 & 15 & 7050 & 734 \\
6023 & 55 & 161 & 0 & 0 & 674 & 173 \\
3 & 5 & 0 & 275579 & 1111 & 1521 & 3955 \\
11 & 13 & 8 & 2176 & 11943 & 731 & 457 \\
20317 & 3920 & 48 & 234 & 46 & 109071 & 17977 \\
1240 & 157 & 2 & 1542 & 443 & 66614 & 43470 \\
0 & 0 & 0 & 730 & 133 & 4329 & 19544 \\
1572 & 13 & 1 & 8406 & 1476 & 75171 & 142338
\end{array}
\tag{2}
$$

In Eq. 1 and 2 we show detailed breakdown of how the three different algorithms defined SS. In both equations the rows correspond to DSSP 4 categories and the columns to DSSP and STRIDE categories in Eq. 1 and 2, respectively. Hence, the matrix element i, j ($i =$ rows, $j =$ columns) counts how many residues that were categorised as i by DSSP4 were

categorised as j by DSSP or STRIDE.

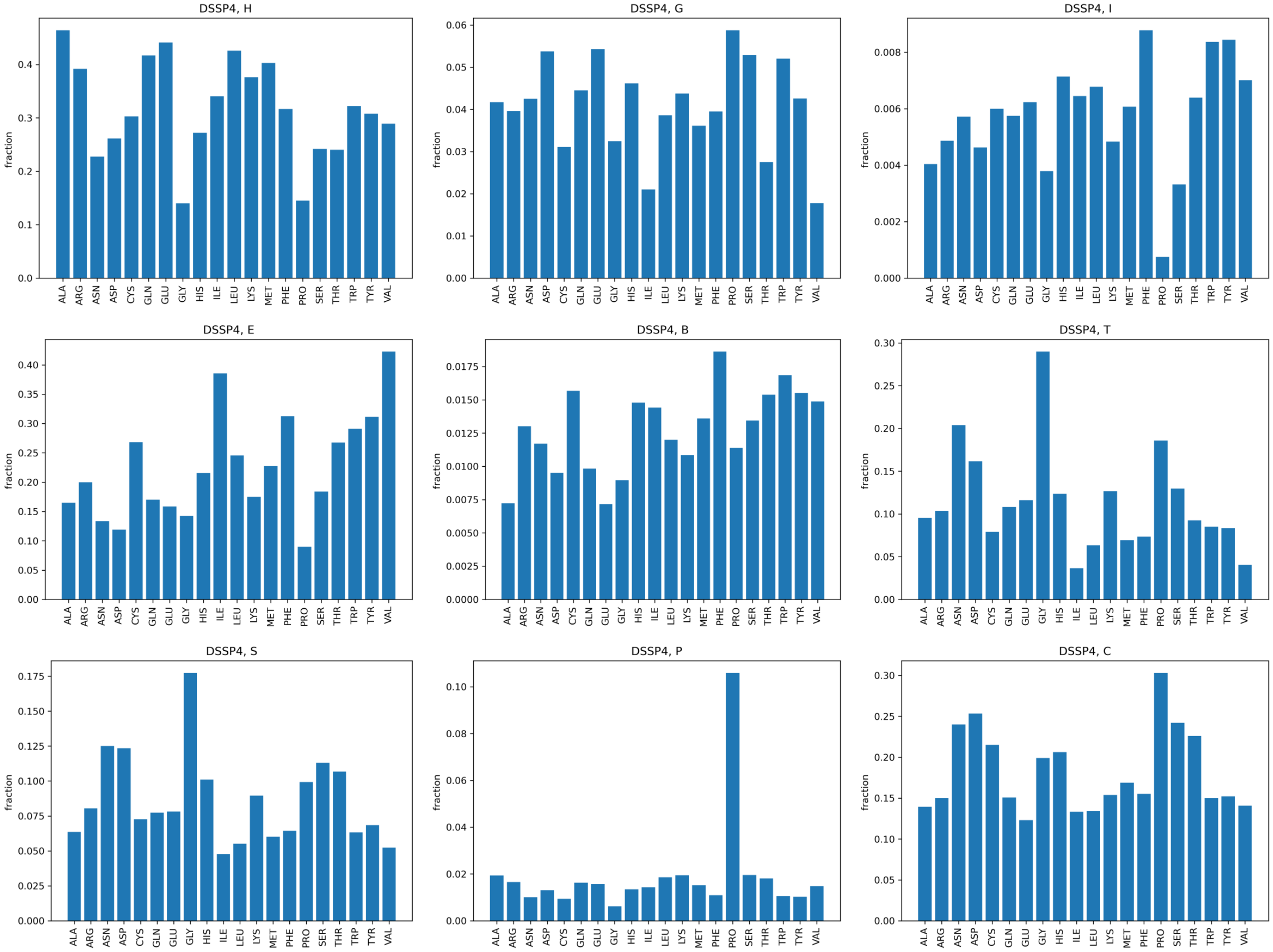

Figure S 3: Bar plots indicating the fraction expressing how many instances of each amino acid type (Ala, Arg,...) belong to given SS category. E.g. we had 106 979 Ala residues (categorised by DSSP 4) of which 49 641 were in category H (by DSSP 4). Hence, the bar in the "H plot" represents 49 641/106 979=0.464. The sum of all bar heights for a given amino acid type is 1 across all plots.

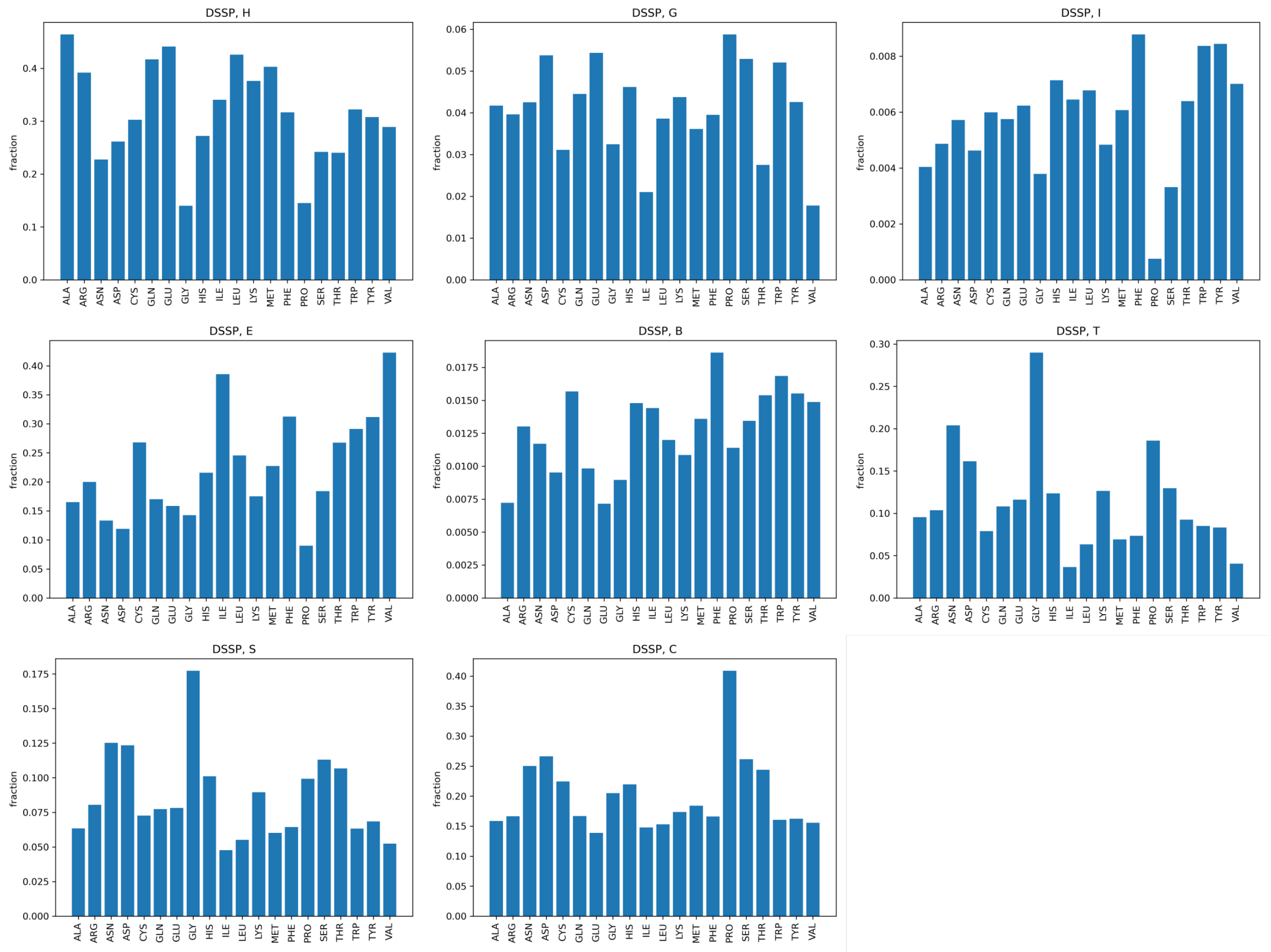

Figure S 4: Bar plots indicating the fraction expressing how many instances of each amino acid type (Ala, Arg,...) belong to given SS category. E.g. we had 106 979 Ala residues (categorised by DSSP) of which 49 641 were in category H (by DSSP). Hence, the bar in the "H plot" represents 49 641/106 979=0.464. The sum of all bar heights for a given amino acid type is 1 across all plots.

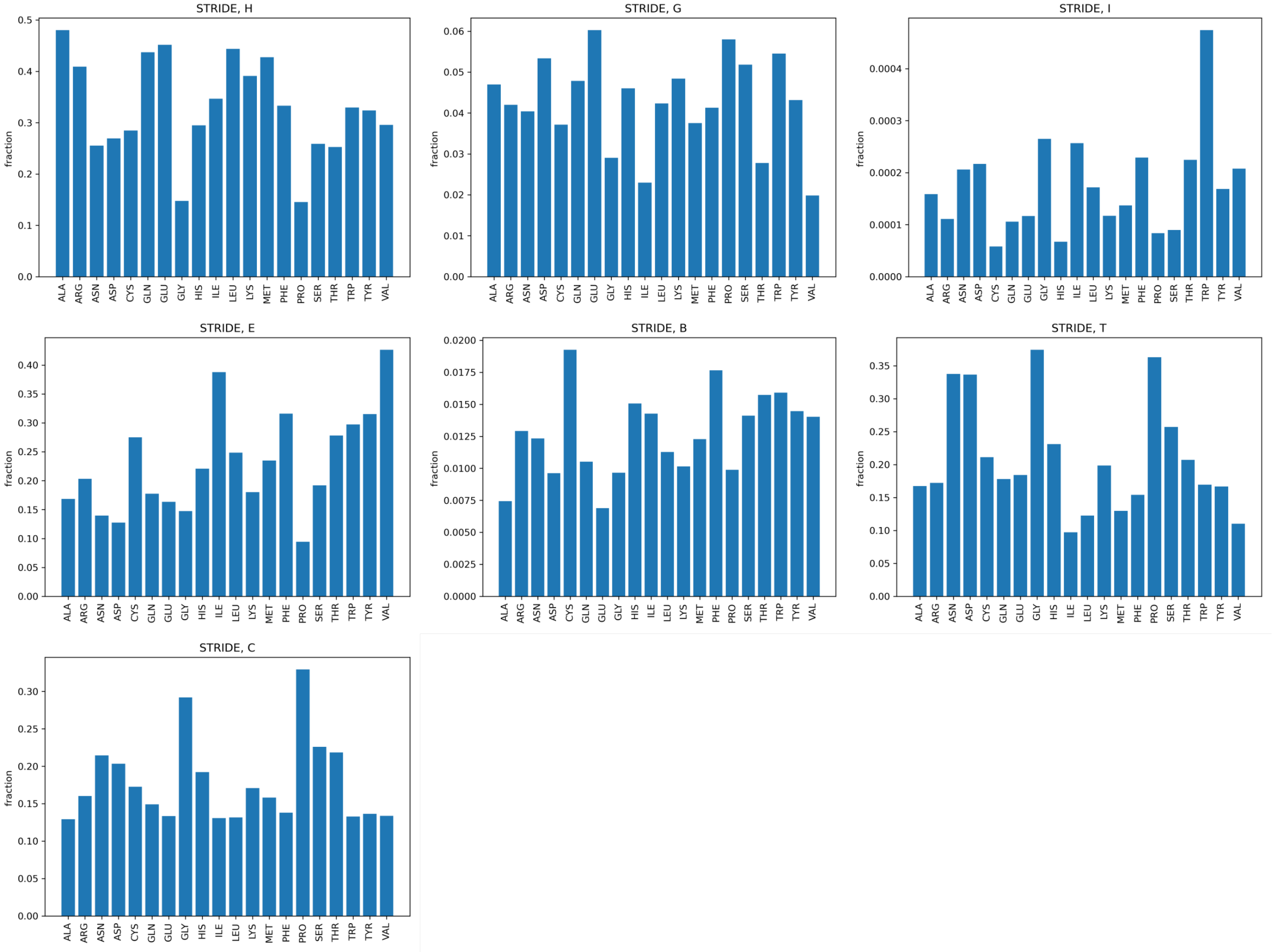

Figure S 5: Bar plots indicating the fraction expressing how many instances of each amino acid type (Ala, Arg,...) belong to given SS category. E.g. we had 106 950 Ala residues (categorised by STRIDE) of which 51 351 were in category H (by STRIDE). Hence, the bar in the "H plot" represents 51 351/106 950=0.480. The sum of all bar heights for a given amino acid type is 1 across all plots.

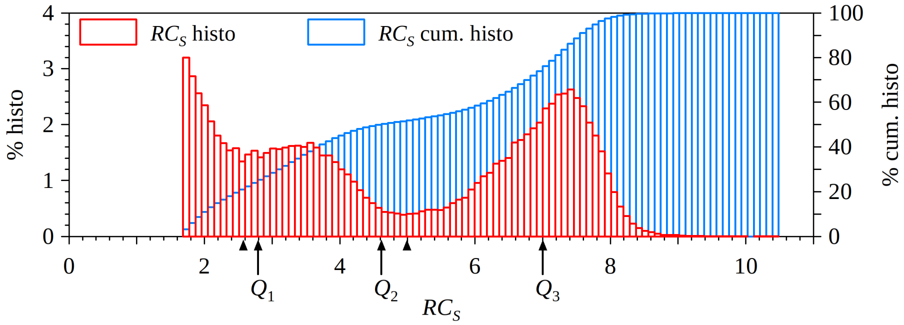

Figure S 6: Histograms of the RC_S distribution for the SS category S as defined by DSSP 4. The quartiles are indicated by arrows, local minima by triangles.

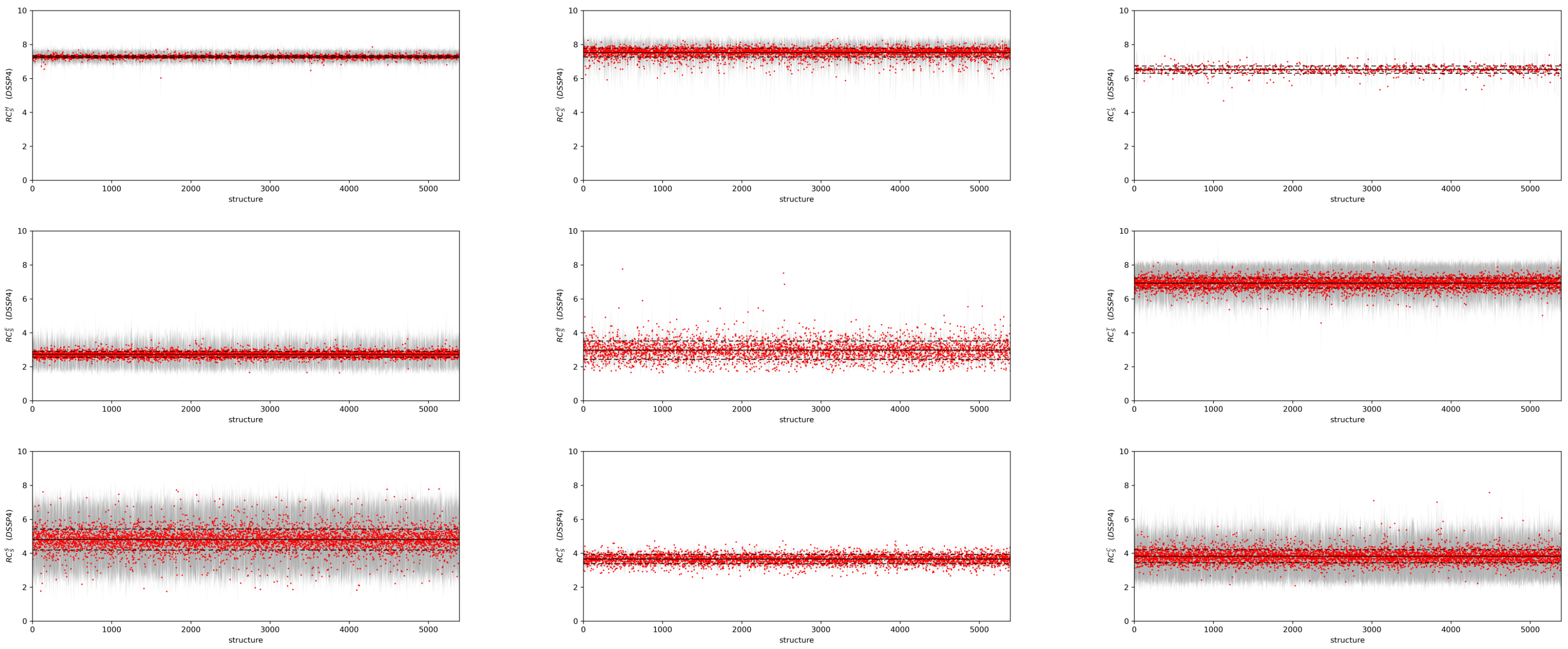

Figure S 7: Average RC_S value per DSSP 4 SS category for each of the 5 392 PDB structures (dots). The average of the averages is marked by the solid line and the average $\pm$ (sample) standard deviation interval is within the dashed lines. The grey area represents the (population) standard deviation within each PDB structure.

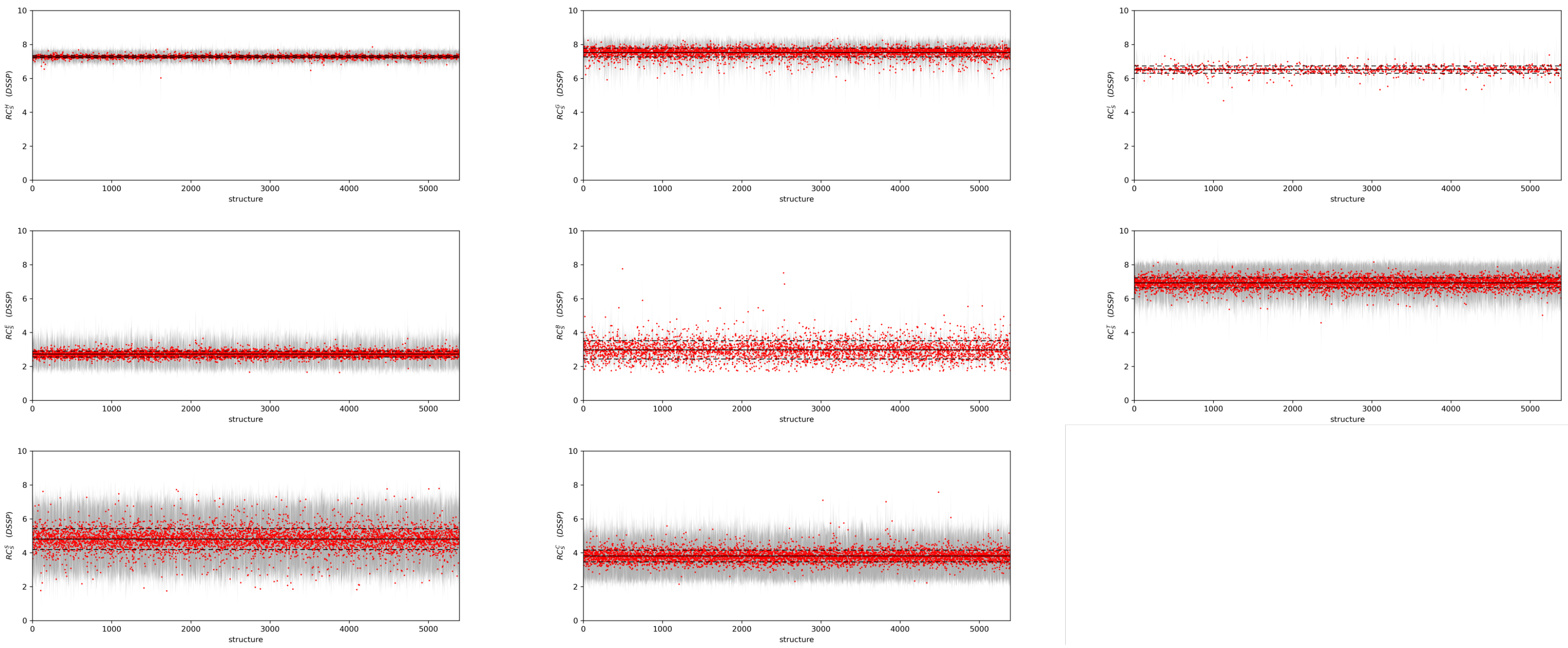

Figure S 8: Average RC_S value per DSSP SS category for each of the 5 392 PDB structures (dots). The average of the averages is marked by the solid line and the average ± (sample) standard deviation interval is within the dashed lines. The grey area represents the (population) standard deviation within each PDB structure.

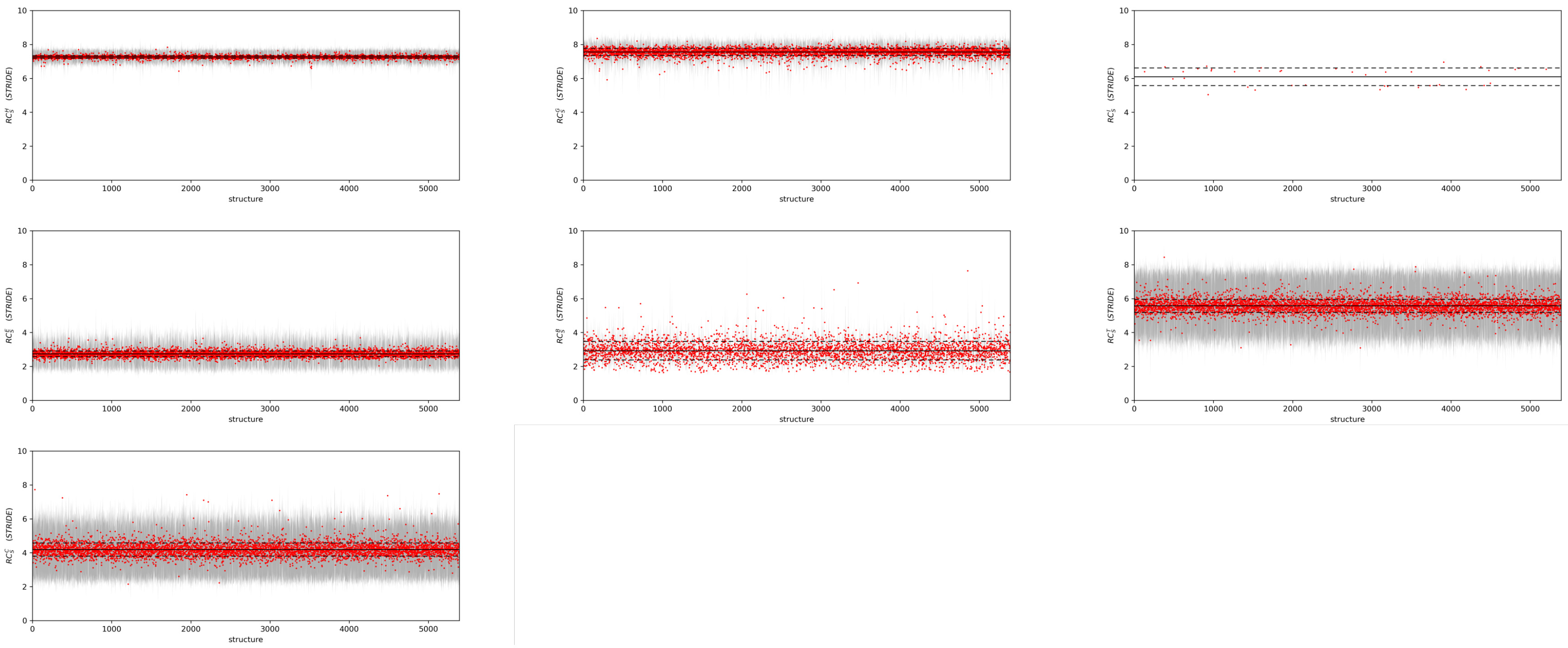

Figure S 9: Average RC_S value per STRIDE SS category for each of the 5 392 PDB structures (dots). The average of the averages is marked by the solid line and the average ± (sample) standard deviation interval is within the dashed lines. The grey area represents the (population) standard deviation within each PDB structure.

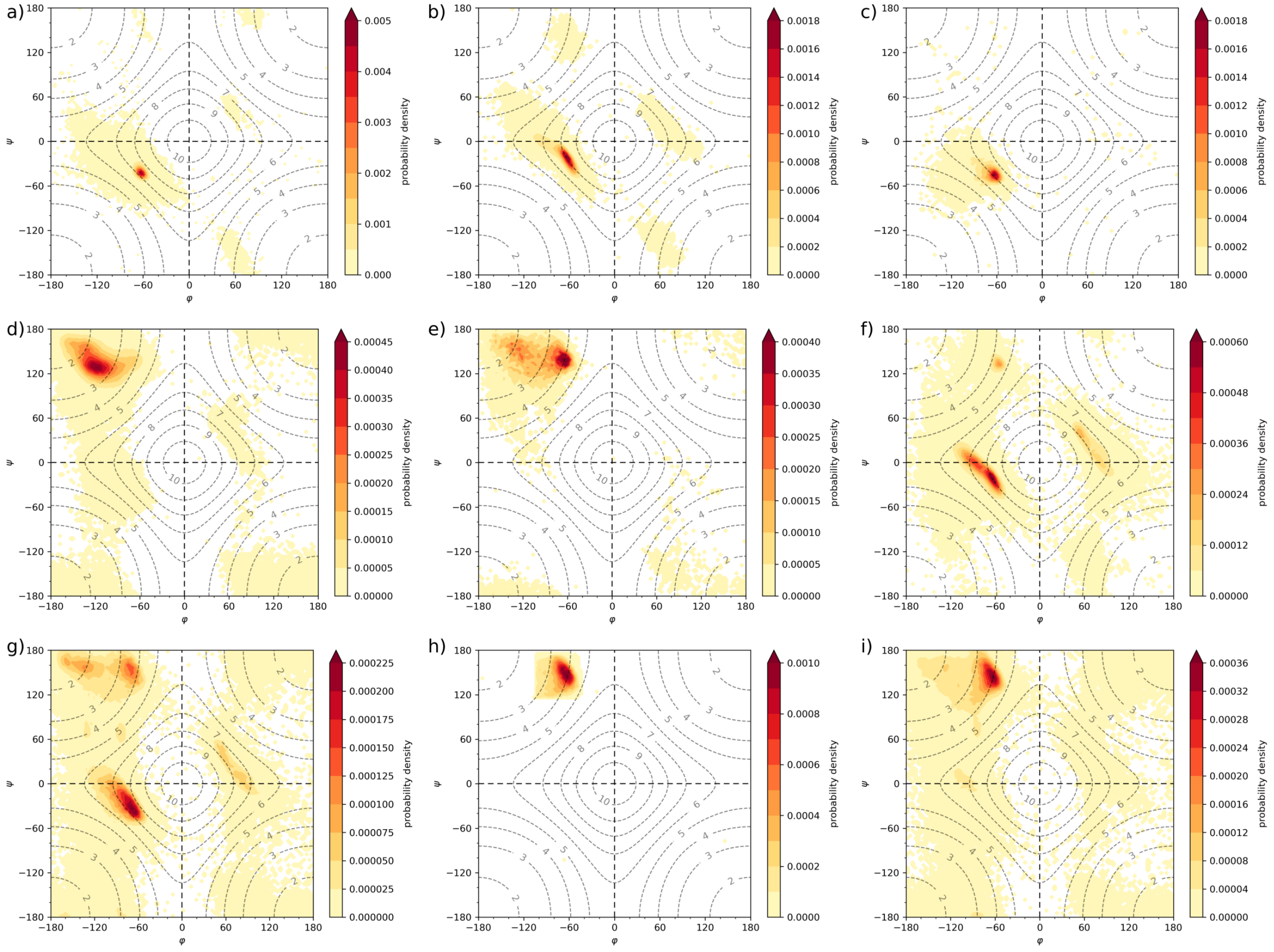

Figure S 10: Ramachandran-like probability density histograms for residues classified as a) H, b) G, c) I, d) E, e) B, f) T, g) S, h) P, i) C by DSSP 4. The theoretical RC_S values are displayed as contours. (Histograms are normalised so that the sum over all grid points yields unity.)

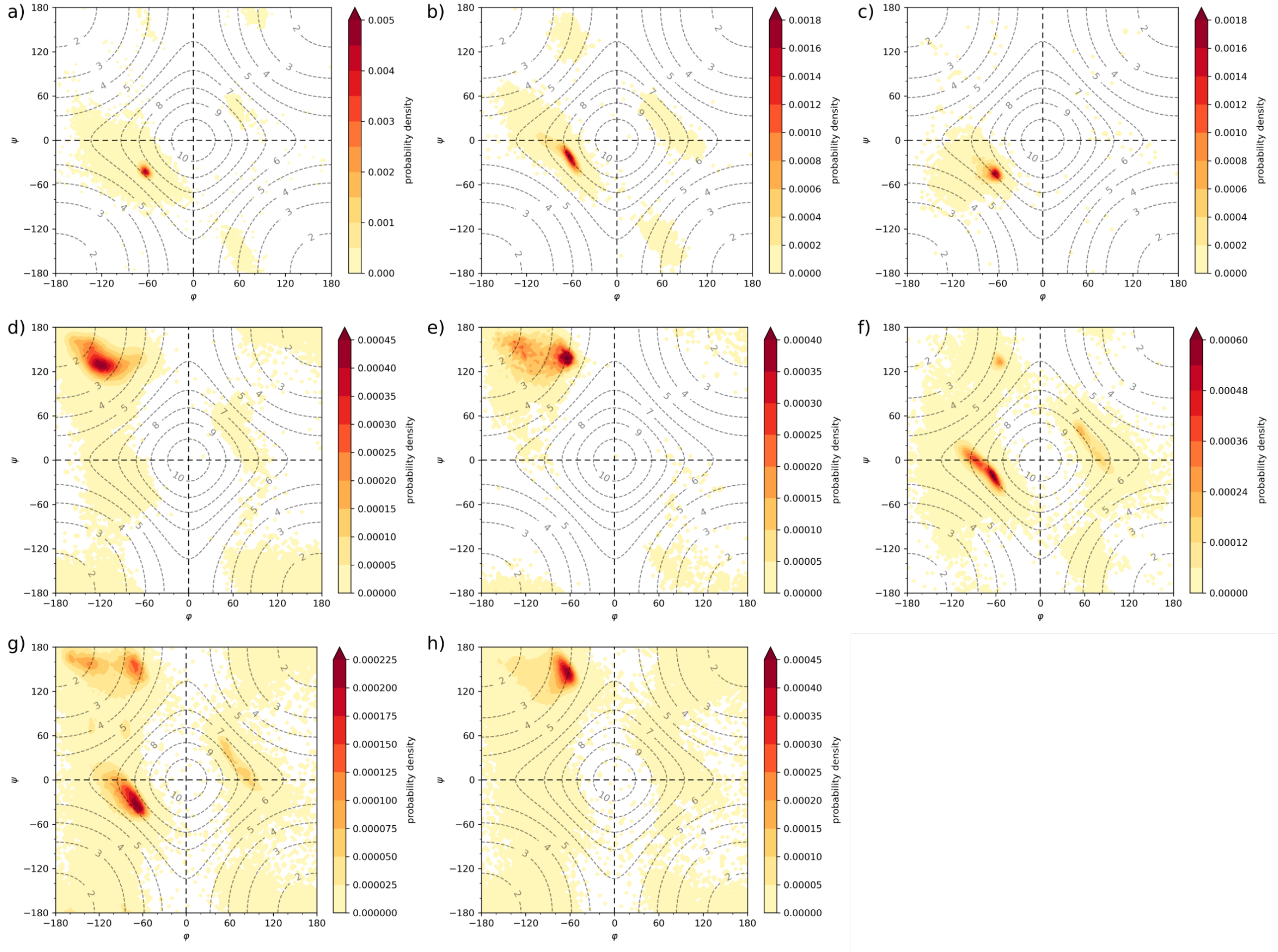

Figure S 11: Ramachandran-like probability density histograms for residues classified as a) H, b) G, c) I, d) E, e) B, f) T, g) S, h) C by DSSP. The theoretical RC_S values are displayed as contours. (Histograms are normalised so that the sum over all grid points yields unity.)

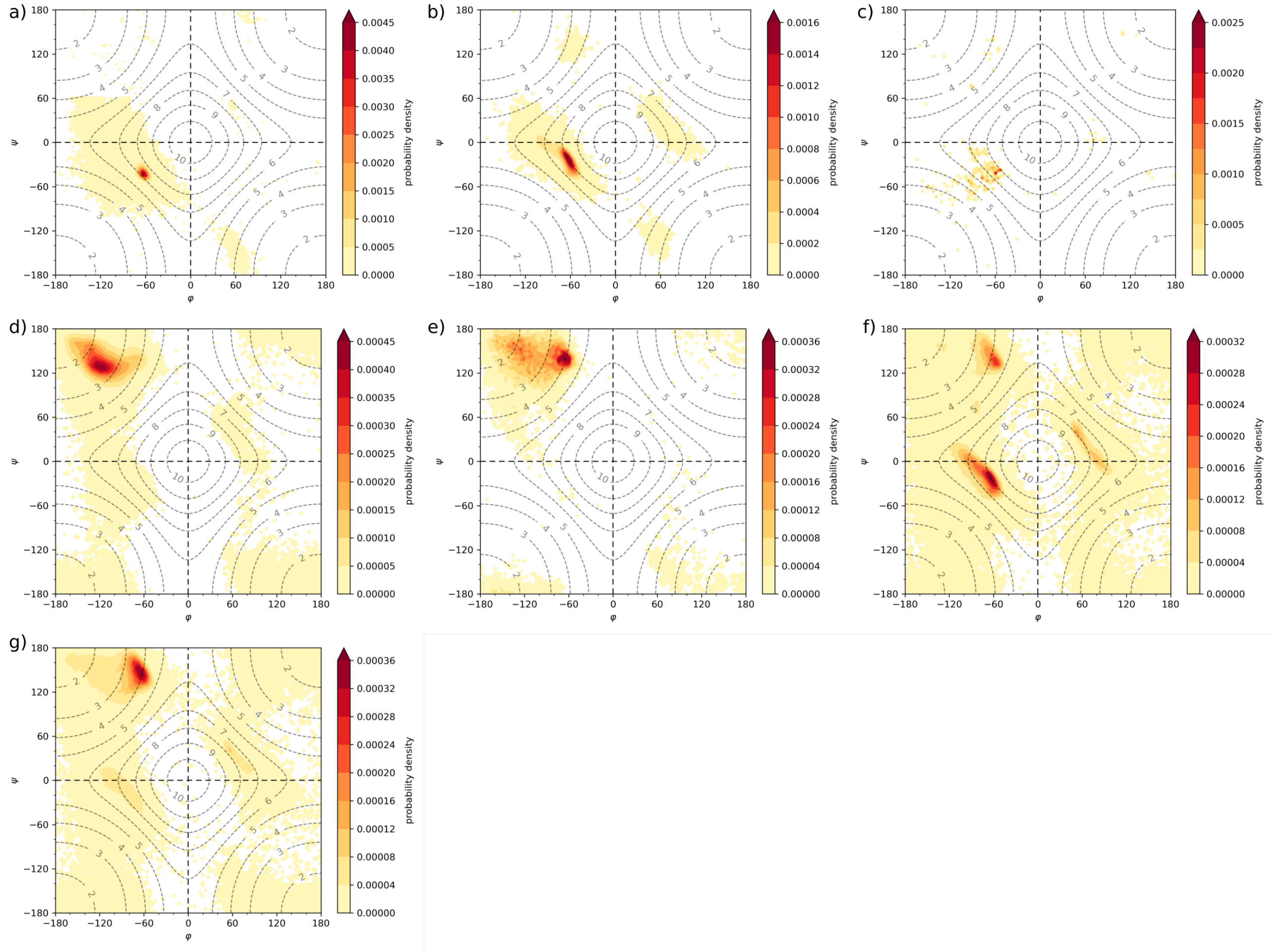

Figure S 12: Ramachandran-like probability density histograms for residues classified as a) H, b) G, c) I, d) E, e) B, f) T, g) C by STRIDE. The theoretical RC_S values are displayed as contours. (Histograms are normalised so that the sum over all grid points yields unity.)

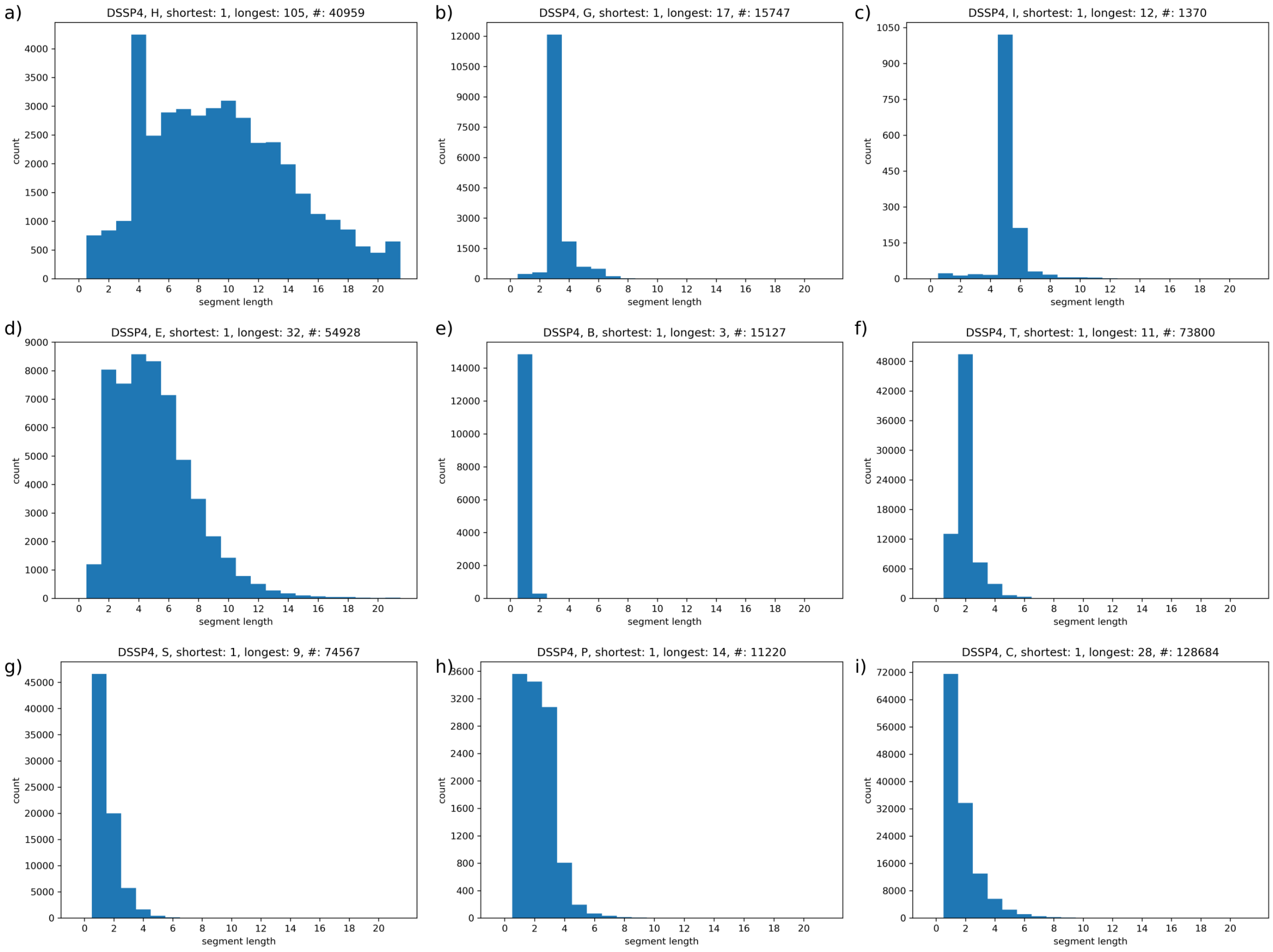

Figure S 13: Histograms of lengths of secondary structure motifs. Herein a motif is a consecutive series of residues classified as one SS category by DSSP 4 a) H, b) G, c) I, d) E, e) B, f) T, g) S, h) P, i) C. The shortest and longest motif length is written out, as well as the number of motifs in given SS category in the set of used PDB structures.

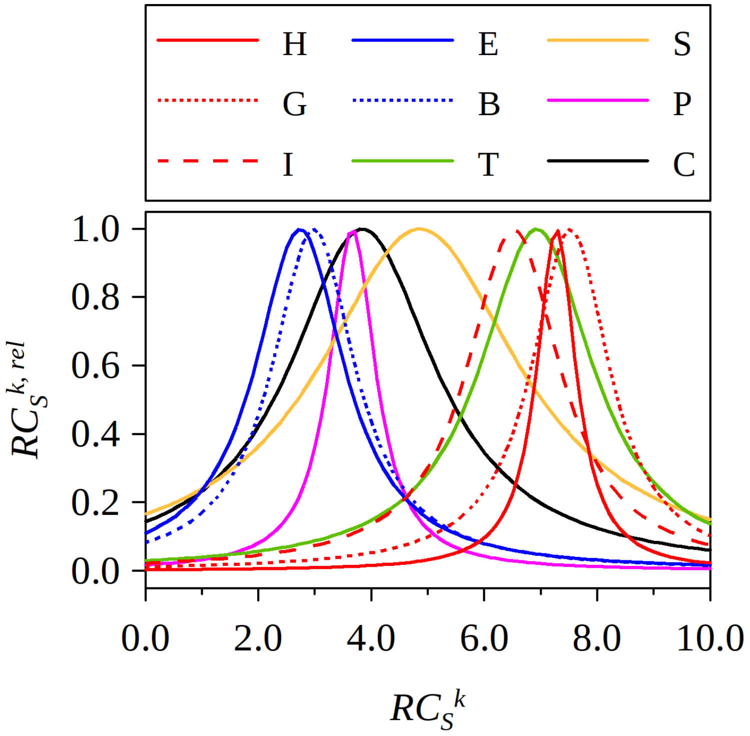

Figure S 14: The theoretical dependence of the relative residue folding degree for each SS category (as by DSSP 4). The $RC_S^{k,rel} = \frac{1-\left[\left(RC_S^k - RC_S^{ref}\right)/RC_S^{tol}\right]^2}{1-\left[\left(RC_S^k - RC_S^{ref}\right)/RC_S^{tol}\right]^4}$ with RC_S^{ref} and RC_S^{tol} for each SS category as listed in Table S 1.

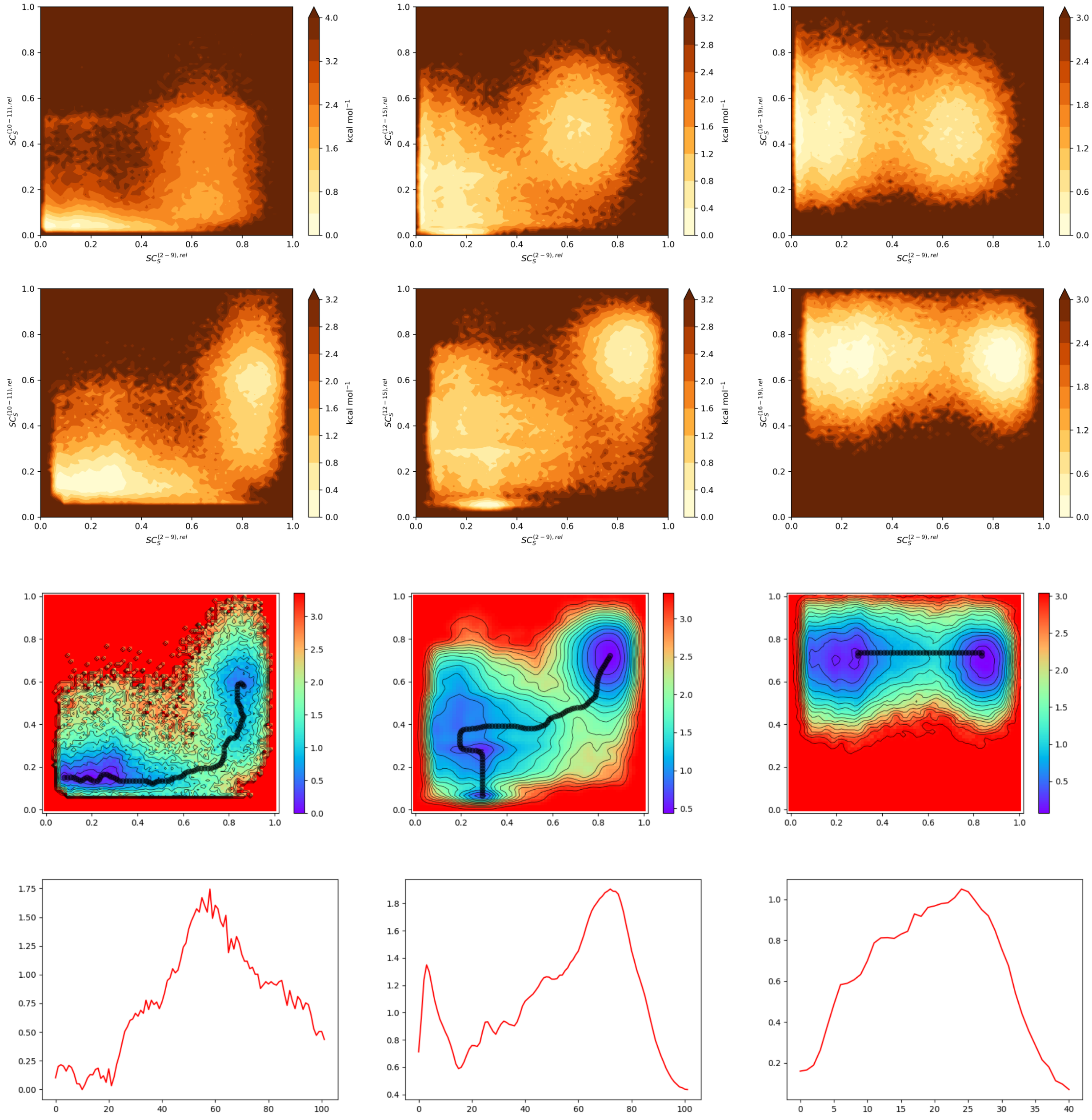

Figure S 15: Potential of mean force (PMF) surfaces (a.k.a. free energy landscape, FEL) at 300K for the folding process of the Trp-cage protein captured by four CVs. The mean RC_S in given segment in given reference structure was used in each respective segment as RC_S^{ref} for each residuum in the segment (see Figure 5 in the main text). The coordinate $SC_S^{(2-9),rel}$ is always on the horizontal axis and on the vertical axis are $SC_S^{(10-11),rel}$ (first column), $SC_S^{(12-15),rel}$ (centre column), and $SC_S^{(16-19),rel}$ (last column). The top PMFs were constructed with $RC_S^{tol} = 0.5$, the lower ones for $RC_S^{tol} = 1$. The resulting minimum energy paths (MEP) between the lowest minima are shown. The energy profiles along the MEP coordinates are shown as well. All energies in kcal mol^{-1}.

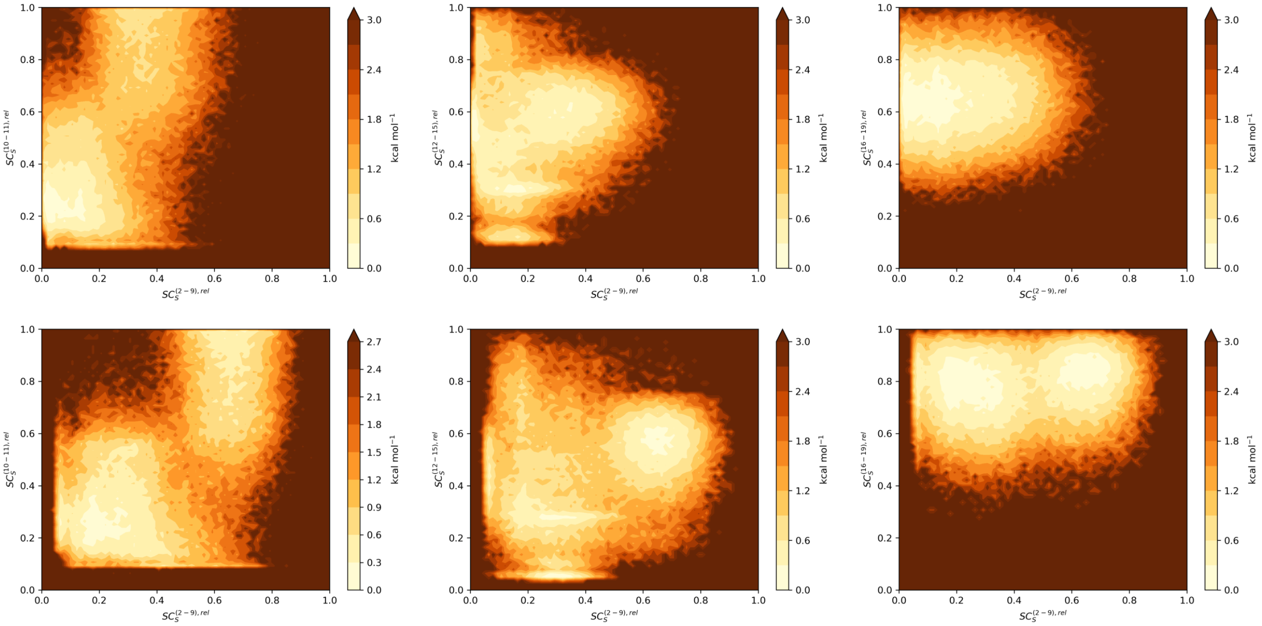

Figure S 16: Potential of mean force (PMF) surfaces at 300K for the folding process of the Trp-cage protein captured by four CVs. The RC_S^k of each residuum in given segment in given reference structure was used in each respective segment as RC_S^{ref} for the corresponding residuum. The coordinate $SC_S^{(2-9),rel}$ is always on the horizontal axis and on the vertical axis are $SC_S^{(10-11),rel}$ (first column), $SC_S^{(12-15),rel}$ (centre column), and $SC_S^{(16-19),rel}$ (last column). The top PMFs were constructed with RC_S^{tol} equal to the standard deviation of RC_S for the DSSP 4 category to which each residuum belongs in the reference structure (see Table S 1), the lower ones for $RC_S^{tol} = 1$. All energies in kcal mol^{-1}.

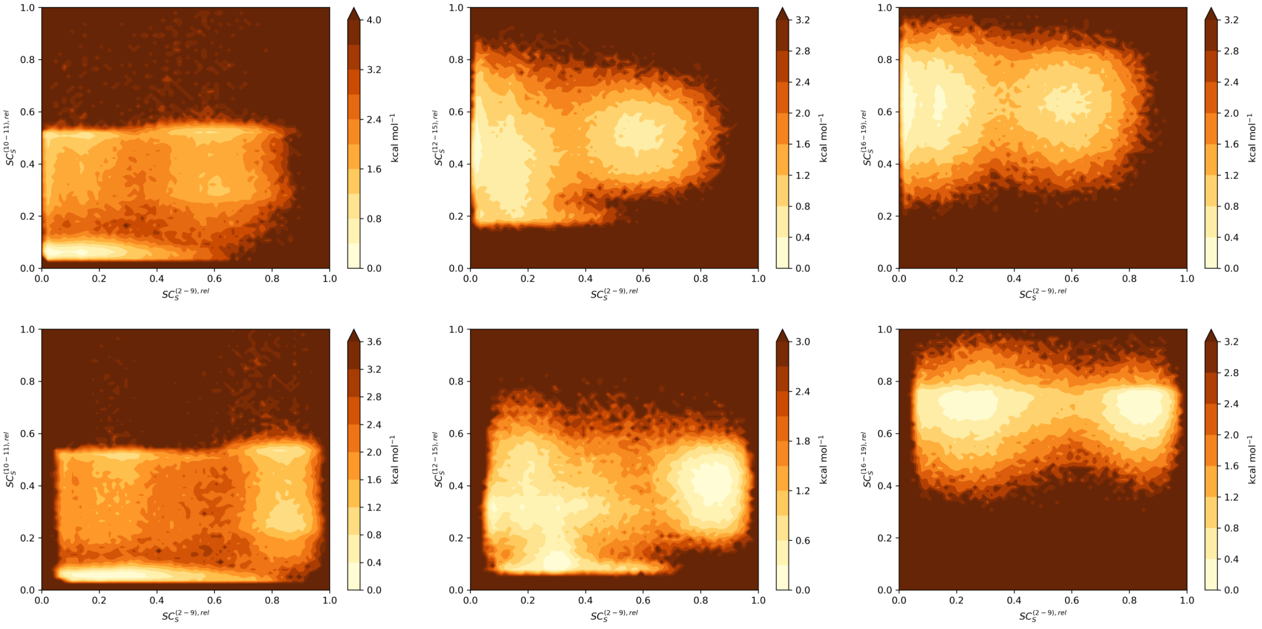

Figure S 17: Potential of mean force (PMF) surfaces at 300K for the folding process of the Trp-cage protein captured by four CVs. The typical RC_S of each DSSP 4 category of each residuum in given segment in given reference structure was used in each respective segment as RC_S^{ref} for the corresponding residuum. The coordinate $SC_S^{(2-9),rel}$ is always on the horizontal axis and on the vertical axis are $SC_S^{(10-11),rel}$ (first column), $SC_S^{(12-15),rel}$ (centre column), and $SC_S^{(16-19),rel}$ (last column). The top PMFs were constructed with RC_S^{tol} equal to the standard deviation of RC_S for the DSSP 4 category to which each residuum belongs in the reference structure (see Table S 1), the lower ones for $RC_S^{tol} = 1$. All energies in kcal mol^{-1}.

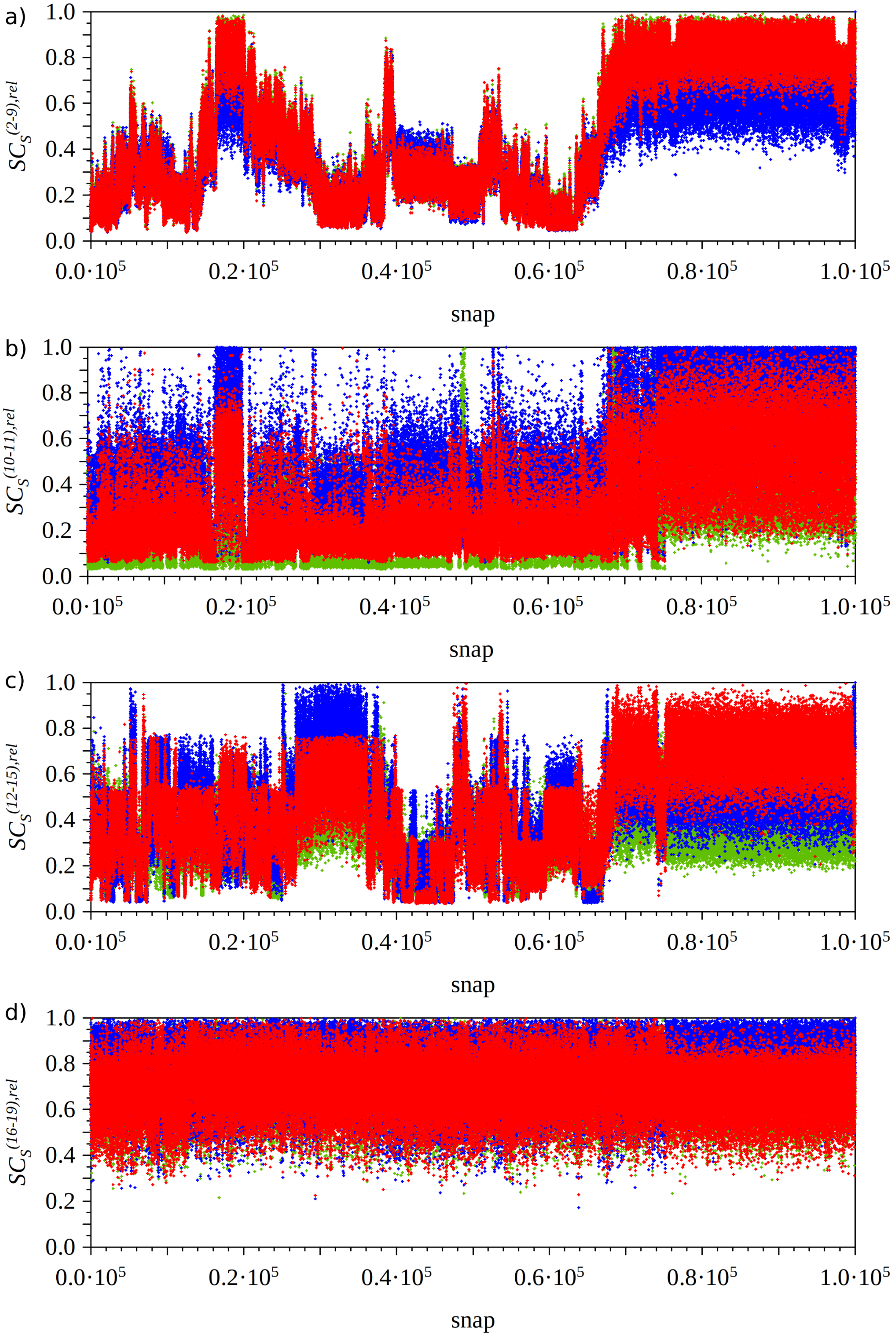

Figure S 18: $SC_S^{\kappa,rel}$ values for different reference RC_S^{ref} values; red: average RC_S in segment, blue: individual RC_S of each residuum, green: characteristic RC_S for each category of each residuum in the reference structure. All values are for $RC_S^{tol} = 1$. a) $SC_S^{(2-9),rel}$, b) $SC_S^{(10-11),rel}$, c) $SC_S^{(12-15),rel}$, d) $SC_S^{(16-19),rel}$.

References

(1) Estrada, E. Characterization of the amino acid contribution to the folding degree of proteins. *Proteins* **2004**, *54*, 727–737.

(2) Sladek, V.; Harada, R.; Shigeta, Y. Protein Dynamics and the Folding Degree. *J. Chem. Inf. Model.* **2020**, *60*, 1559–1567.

The structures which can be accessed from ccPDB 2.0 under the name PolyprOnline, or the authors' web page https://www.dsimb.inserm.fr/dsimb_tools/polyproline/about.php were used. The dataset contained some 24 761 folded protein structures of which these 5 392 pdb structures were successfully used, i.e. could be downloaded, DSSP 4, DSSP, and STRIDE calculation finished without errors and subsequently the RC_S calculation finished without errors as well.
This is a list of the PDB IDs of the used structures:
2qzu, 2bib, 2fgq, 1ash, 1ail, 3tl2, 2h01, 2icc, 1dfu, 3elw, 2fi1, 1qua, 3ik2, 3fmu, 2v3i, 3nwo, 3zq5, 1uxt, 1hkf, 3ksv, 1vgj, 1mj5, 3fb4, 1j99, 3ozp, 1x3l, 2yhg, 1nu5, 3gx0, 2cf5, 2zp1, 3c3g, 1fna, 1zva, 3hra, 3q8k, 3dlm, 2wq7, 2yh5, 3chj, 2rku, 4gb5, 1g1t, 1sb8, 3axb, 1npc, 1m4m, 3f67, 3asa, 4aqo, 1f4p, 2ei9, 2w47, 2pn6, 4ipf, 1qto, 2wyq, 3neu, 1edz, 2jln, 3giw, 2ea3, 1a7w, 3ign, 3emv, 2osa, 1okc, 3fsd, 3nul, 1h2e, 3gx8, 2pia, 3rle, 3uw1, 2d5, 2qa2, 2igv, 1roa, 3dmi, 3mx7, 3aks, 2nml, 4gey, 3hdp, 1u61, 1q20, 2uyt, 3eg3, 1t1u, 3hbk, 2qy, 3sol, 3h7r, 3iuf, 2iug, 1qhv, 3ege, 3eye, 3g7p, 2oy, 2ymv, 1kq3, 2ra9, 2q9k, 3b7h, 1pii, 2jh1, 2z00, 1g94, 4fdw, 3f43, 2vap, 3mql, 3o7, 2z0x, 1vku, 1srv, 2fzl, 3su6, 2v03, 3gjw, 1pi1, 1hq0, 1a32, 2w4f, 3oru, 3dr3, 3oeq, 3mu7, 1syy, 2gjl, 1php, 3msw, 3zh9, 1es5, 3hzp, 2c78, 1b0u, 1p1l, 1xfj, 1ydf, 4gh, 1cs6, 6rx, 3f6, 3u26, 2ot9, 3frr, 1ntn, 3tnt, 1sra, 2e2o, 3zs3, 2vuw, 3cc8, 1qwk, 1ln1, 1i71, 1g62, 1s3c, 2j8b, 3h0x, 4ex6, 3ufi, 3n9t, 1crb, 4es6, 4fz4, 1m4l, 1lmq, 3ujc, 4h2g, 3hc7, 3o9j, 3dau, 3ov8, 2qsa, 1vct, 4tmk, 2pmr, 3q8g, 1wni, 1g6h, 3s1s, 2b1m, 3ff2, 1wza, 2gh9, 1by, 3u55, 3llb, 3dha, 2bcq, 2qhf, 3hf7, 2yc, 2o03, 2z81, 2qgu, 1a1x, 2ets, 1o13, 3gs3, 1c6r, 3a03, 1c96, 3a04, 2b8m, 3u0v, 3zqx, 1xu, 2h85, 3v1a, 2zcx, 1vmj, 1poa, 1rh9, 3is3, 1zx6, 3q4o, 3im8, 3i47, 1zmr, 1a17, 3piw, 3fmy, 3pl1, 2pbp, 2d4, 3gbl, 1hmt, 1pja, 1g6s, 3jte, 3rd2, 1lqy, 2dru, 1tif, 3mtv, 3h7m, 3mpy, 4dca, 3eaz, 1xph, 3ch0, 1ixh, 1gai, 4jw, 3u7v, 1wos, 2w7t, 2d9r, 1xtz, 2wzo, 3ga3, 3e8s, 3tqk, 2wq, 2id6, 3otg, 3tjy, 2c6u, 3qrl, 7pcy, 2gpi, 1co6, 3amr, 1zxx, 1ru4, 1mz4, 1tmo, 3li9, 1gnt, 3r6d, 1jm1, 1enf, 1ji6, 1qoi, 3ejk, 1j0p, 2dbo, 2b2h, 1iyn, 4aw0, 3r6f, 2vge, 2bqq, 3lhh, 1puc, 2w8t, 1bx4, 1o4v, 2oeb, 2ph1, 2nsa, 2ogg, 2czw, 1zhv, 3na7, 3ne7, 2qti, 1ah7, 4fiw, 1zk9, 3a2, 2y0o, 1ijb, 1zyl, 2die, 1fmb, 1kzl, 1t3y, 2a4v, 3p6l, 1gqv, 3pfz, 1jh, 1i3j, 2j49, 1vi4, 2isb, 2z2n, 3b0g, 4hbs, 2z8l, 2apr, 2en, 2vov, 2bue, 3hdl, 3l60, 1u02, 2g7b, 2r9i, 3i45, 3ti9, 3bnj, 1gkm, 4kq, 4i19, 1x3o, 1coj, 3u5s, 3rgk, 3hui, 3f7w, 3n9k, 2g7s, 4i90, 2b0a, 3k7x, 2qo4, 3u9r, 1ci, 3byd, 2qyu, 3rlo, 4et7, 1lfp, 2x4l, 2xu3, 2xy, 1yht, 1lwb, 1hfc, 3teu, 2yw, 1w78, 3hny, 1vlb, 2bqx, 4f67, 2pr, 1h6t, 4ddj, 3vxv, 3lr, 3hf0, 2e87, 1xkr, 2ije, 2ale, 2rfr, 7a3h, 2wl1, 2ozt, 1w0h, 1dtz, 1oz9, 3fh1, 3n6m, 3ct5, 2cve, 2is, 4idl, 4hf7, 3suj, 3o7b, 4gq7, 1vjg, 3on1, 2fo3, 1fyv, 1saz, 4eic, 3sft, 1ub9, 3dwo, 1dqg, 3tvq, 1wcu, 3e0h, 1iro, 3cfw, 1c3g, 3hhv, 3w07, 2r16, 3gna, 2wn, 1j8, 1s29, 3kbb, 1vhu, 4i17, 4gky, 1k7j, 2r01, 3k63, 3mbr, 1kbl, 1lht, 1aep, 4h4, 2p9t, 3or5, 4hyq, 4af1, 3bo, 1ppo, 2pm, 1ytq, 2x7m, 2nnj, 1mxs, 3arx, 4f25, 3q, 1edg, 1dtx, 1gm6, 2xp1, 3k6i, 2p0, 3k1u, 3agn, 4dw1, 3h3e, 1i8o, 3mdu, 2wcj, 1ldf, 1trb, 2lis, 1i6a, 4jqr, 1jhd, 2xhi, 3kw8, 2yw8, 2xzz, 2qen, 3fdr, 3hwu, 1wv3, 4hdj, 3tq5, 3qv, 3bia, 2q3p, 4je, 1ym5, 1jpc, 2jc5, 3g9x, 1ng6, 3emn, 2wol, 1rrh, 3br8, 2x1f, 2apx, 3i99, 2r9v, 3r0n, 1v7r, 1lk, 3rfj, 1qrp, 4a05, 1ako, 3e4h, 2bk9, 1ufy, 2vla, 1tg0, 3h4x, 2azq, 1x8y, 3q2c, 2v84, 1svy, 2cv, 2hmc, 1t00, 2vq8, 1jyk, 2x5y, 2o8, 1t1g, 4ig, 1k77, 2j6b, 2p3h, 1gc5, 2iqt, 1oyg, 1bu, 1vpq, 2hr, 2yln, 1wol, 3hms, 2fhf, 2qoj, 2x8h, 3c8f, 1lfw, 1p9i, 1cr, 3tm0, 3kht, 2pwa, 2nwd, 3gwm, 2wfb, 1r3s, 1iuk, 3gmi, 2v7y, 1n4w, 1bg, 2qnl, 4gco, 2ig, 3p54, 1gvh, 3og2, 3oio, 1im5, 3rhb, 2v6g, 3gn, 2qpz, 1m9i, 3ux1, 2v3a, 3zux, 1us5, 3kff, 4arh, 2g6f, 3i7t, 1zkz, 3p9c, 2ev, 3a4c, 2gxq, 2yfo, 3hjh, 3tu6, 2i24, 2wzl, 3hak, 1u53, 3c8y, 3ctz, 2ptm, 1svs, 3aal, 1yck, 1u3i, 3kos, 2jex, 1r6j, 4dfk, 3gd6, 1fr, 4er, 1fa2, 2ja9, 3ga2, 1vk1, 2wan, 1pi2, 1a8s, 3vp5, 3w5, 1dkq, 1gny, 1t5j, 2fu2, 2ilk, 2bnh, 1j72, 1cjw, 1r8e, 3tny, 3goe, 3n5a, 2riq, 3r87, 1p4t, 3ca7, 1zd9, 1b4, 4i16, 3kzn, 2vh7, 1pzw, 3i2k, 3l1x, 2o5r, 1rwj, 1v72, 2x9k, 3twy, 1y9l, 1unq, 1pdr, 1rq, 2na, 6pax, 3bqy, 1pm1, 1su0, 4dny, 1dmr, 3g4p, 1i4w, 1ia, 2pnn, 3zu3, 1pfo, 3adg, 3bpv, 3bj5, 1pmy, 3g2b, 1i1n, 1iap, 2wlr, 1brt, 4afm, 3m3p, 2ppn, 1v05, 3a4j, 4a56, 3h0n, 3pu5, 3ebv, 3uyq, 2oa2, 1rhc, 4i68, 3fw9, 4agh, 1gv, 4il7, 1yqs, 1zrn, 3lop, 2a14, 2frg, 2p2r, 3vy8, 1r6n, 2oc5, 1vf1, 2huo, 3k11, 1oth, 2ek, 3i10, 3ngw, 4hwm, 2je7, 2f60, 1u2p, 2cyj, 3d1, 1g12, 3frg, 1c44, 2nx8, 2no2, 3l8, 3i8b, 1s7i, 4fca, 3m3m, 1nr0, 2igp, 2yex, 1yat, 3a09, 1lam, 3llc, 2vw8, 1yna, 1j8m, 3gv0, 2pet, 1ra6, 2iwr, 3jv1, 3cxm, 3se, 1mr3, 1xov, 3us4, 3m8u, 1tke, 3s4e, 3pbf, 1i60, 3s5o,

3g7m, 3p2j, 3ctg, 1em2, 1k92, 2bkf, 3e5, 2v1m, 2vg9, 2ux7, 3bci, 1kr4, 3f3b, 2por, 3h0o, 1b9o, 3fkc, 1lln, 2d62, 1tg7, 3gwi, 1ezj, 4akf, 2bi7, 2ww5, 2c2j, 3lx3, 1t6a, 1h0, 2qpw, 2gj, 1n00, 1j2z, 1nfj, 1l3p, 1rgy, 2hsb, 2e28, 2r48, 3eif, 2yxx, 1sbx, 3o6p, 3rzv, 2r2i, 4fs, 3vmj, 3pvg, 2v1p, 2o6r, 3i0, 3pz7, 1uas, 2cws, 2b1k, 1x3k, 1fw8, 1o70, 4asc, 3vla, 3uu, 3f7m, 3a38, 3oms, 1jf, 3ezl, 3f6y, 4f8z, 1is3, 2dpw, 2grc, 4jxj, 2b8i, 4f78, 3r6u, 2gou, 3dfg, 3ilh, 3kp8, 1qj8, 1ikt, 1fd9, 2wm1, 2wsk, 1u09, 1mlw, 3go2, 3qt9, 1y9q, 1l8f, 1tl2, 2wbf, 3c2i, 1tua, 3st8, 3sz3, 2gqw, 4gbm, 4b89, 3aw5, 2aex, 2rkn, 4di9, 4iwx, 1tfe, 2fx5, 2bmm, 2a1v, 1k3i, 4htm, 1u8, 2ebb, 3m9w, 1knb, 3iox, 3gm5, 4e6y, 3rwx, 2rct, 3a2z, 4f3j, 2wu8, 1j8b, 3qoy, 2g2, 1udh, 1u29, 2ysk, 3zq7, 2cz2, 3t7v, 3ipc, 1ly1, 1snb, 2xy2, 3mp2, 1tc8, 1vbi, 1jx6, 3rdy, 2wfg, 4fak, 2axq, 3qv2, 3g0k, 3tsv, 2e56, 4itq, 1a6m, 1w7c, 2q3b, 2f2, 3mj6, 3jw, 3lfp, 2gup, 4fru, 1gwm, 1itx, 2gvk, 4dqa, 2j44, 3e3v, 1nl1, 3ctv, 1s3g, 1h2c, 4b3x, 1off, 3p0r, 3eeh, 2pnd, 3lkv, 3chv, 3okp, 1zi8, 2f82, 1kea, 1y6i, 2wmf, 2bo, 2j5a, 3lqk, 2xwv, 1i9g, 3h9n, 3u3, 1r7j, 1c0, 3ii, 3c2c, 1w0p, 1fnl, 1duw, 2exu, 3qe7, 1hy, 3taw, 1z06, 2x46, 3hg7, 1kmv, 1p4x, 2dt4, 1bvo, 3irv, 4j2f, 1mla, 1rlj, 3hp4, 1whi, 1bxv, 1nxc, 3k7y, 4dc, 2wj5, 3qp4, 1z3q, 1sef, 3qe3, 4evm, 3fy3, 1sf, 1yqb, 3lhi, 4gzc, 4b88, 1knw, 3ffy, 2qed, 4hhx, 2g29, 1lki, 1kqr, 2aqj, 1rgz, 1xqo, 3v86, 1rk6, 2xcl, 4dkj, 3nyc, 1e29, 4db6, 1uuy, 1q6z, 1ag6, 1l8k, 2e1b, 1x82, 4e9f, 1a62, 2qsw, 3ho9, 3t90, 1lpj, 3us3, 1dnl, 2yxy, 1lu4, 1zn, 4iag, 3nm6, 3dr5, 1hl, 4fnv, 2quo, 3w25, 3f4m, 1edo, 3il8, 1exs, 3bv8, 2vfb, 2a3m, 2b61, 1jo8, 2a9o, 2eo4, 1pkp, 1hp1, 1j8r, 1dq3, 3a72, 3vdj, 1gvd, 3pl9, 1n57, 1g4i, 4exk, 1dly, 3ppt, 4ir5, 3lje, 3bt4, 1svb, 1qi7, 2fht, 3hyq, 3zuc, 3o04, 3n17, 3ha9, 2vyo, 1vi, 3eb5, 1xer, 3d4e, 3p6d, 2d0, 1nep, 1f7u, 2ekp, 2bi0, 4ez8, 3q1d, 2znr, 1mbs, 4g55, 1rfs, 1ys1, 1fas, 3ry4, 1tul, 1zx5, 4e2u, 4hs, 2aio, 3kw, 2gee, 2yv, 2xt0, 1qjh, 1j02, 3qww, 2yg5, 1vem, 2qdx, 2bz1, 1yzf, 1w55, 3fnd, 3kwl, 1qr0, 4ar6, 4hm, 4dnh, 2iqy, 2hdo, 3lxd, 1yiv, 3atg, 4dui, 1bg4, 2q3w, 3tss, 2hwk, 4h, 1jln, 1jcf, 1fl2, 1tqh, 3i5x, 1pk, 4axv, 3nvs, 1b0x, 2nsz, 3nqh, 2rdg, 3now, 4g0x, 1qtf, 2bmv, 4e40, 1b63, 1lw, 3ibd, 2sak, 1bgv, 3jtj, 3o26, 1nr6, 2hi4, 2w3j, 3trc, 2xv7, 2d1s, 3emi, 2g5g, 1aj2, 2j3x, 2jhq, 1io2, 1j1t, 1cc, 3fcn, 1qgi, 3rva, 1ouo, 3ofm, 3du1, 2q9r, 1aky, 2hl0, 3q5t, 2oxo, 1qxy, 1kn, 3knv, 3vyx, 4f55, 2xo3, 3fgh, 2re7, 1a8p, 3hfw, 2c9q, 2yvp, 1eg3, 2nyv, 3pg0, 4j4z, 2v4x, 3ke3, 4dw, 3ju0, 2og4, 1dyp, 3nyt, 1ug4, 2y1, 1zkl, 2hk, 4dv8, 1sfe, 1i8a, 3ga8, 3h8n, 3aml, 2hbo, 1gak, 3ios, 2v3g, 2pl5, 1k8w, 3mc3, 2icg, 3ds8, 3n98, 4eys, 3vii, 1gjw, 2ewt, 2jl1, 4atg, 2c7, 1tev, 4kvh, 3bzm, 1lsl, 1fno, 2j0i, 2p5y, 4dww, 1u8b, 1jdw, 4f8l, 4gbu, 2wje, 3tq2, 4ak2, 4ikh, 1sqg, 2pv4, 2okm, 1fyx, 2ckk, 1lr7, 3e7r, 1mwp, 3ndq, 1j93, 2p39, 4fkc, 2hl7, 1p5, 2ok3, 1wf3, 1yk9, 2ims, 2dg7, 4gx, 1s69, 1e4c, 4jhg, 4, 1lr0, 2fwh, 4g26, 3zco, 2yzt, 1yio, 1xfk, 1jfx, 1v77, 1mg4, 2jjn, 4gk6, 1uxx, 3i8z, 1ctf, 8i1, 1r9h, 2aak, 4ad1, 1wfx, 2fbi, 3g85, 2j89, 3fe5, 2d3, 1vpd, 2zqm, 2z2f, 4iyl, 1cy5, 2q4w, 1qcx, 1nwz, 1o4y, 4g3o, 1shu, 3r8q, 2vga, 1ak0, 2vmh, 3ge2, 1utg, 3p2t, 1ytc, 3u, 2yx, 3ccy, 451c, 3tqe, 1o4w, 4alz, 2yay, 3tx, 1dlj, 1gp0, 1ajj, 3uqi, 1dyk, 1i7d, 1q8b, 2bgi, 1vl1, 3px8, 3tip, 1n81, 1vpr, 3i7m, 2o55, 1nox, 2wc1, 2z1j, 3m6c, 1l5o, 1mxg, 3eg4, 1nb2, 1gui, 1n2s, 1cv8, 3ks, 1mai, 1w5d, 3i, 1vhn, 3cax, 1wwc, 3s57, 2a1i, 3rkg, 1v33, 2od5, 2acy, 1kv9, 1mk, 2qor, 4gs5, 2c0h, 1cid, 3e9v, 1us0, 1v0, 1fcy, 3l4n, 3s6f, 3tk9, 3e6c, 1vmb, 3hpc, 1rk, 3c5a, 4abl, 2ft, 3q0w, 1gd6, 2xhg, 1g5a, 2r44, 3egn, 3i36, 1su8, 1yqt, 4drr, 2geb, 3ayq, 1ass, 1wc2, 1skz, 3iog, 2hcz, 2o4d, 1b0a, 3u2g, 1klo, 3hem, 1pzs, 3ju3, 3vss, 3lsw, 2vq2, 1kqw, 3pyc, 3k5j, 4iu0, 4i4q, 3mos, 1ak2, 2yxl, 2fbq, 3hjl, 3ci3, 3ne0, 2nvh, 2z4u, 1mz, 2ayh, 4efi, 1ph, 2ydt, 3vm5, 3k8w, 3voy, 2fuk, 2c4n, 2oqk, 1koe, 4h14, 1q5d, 4j8, 2c0z, 3p06, 3lqb, 4dz1, 3b0x, 2p5k, 1pvx, 2rjo, 1zzk, 2dji, 2z0w, 1nxb, 3rbs, 1kcm, 2p0t, 3pfe, 1q7h, 1xwm, 3cin, 1ks8, 4at0, 1tuk, 2fg1, 1gsa, 3hbz, 3i6v, 1esc, 1bol, 4f6m, 1j1v, 3fwt, 3i28, 4fiv, 1m1h, 2qgg, 3caf, 1qcq, 3f6p, 2wag, 2xo, 1v2z, 1wer, 1i76, 3g91, 2ah5, 3l9a, 2w6k, 3qsd, 1s8n, 2g17, 2o2g, 4ek, 3seb, 3dqy, 1nza, 3qzx, 1pea, 2xym, 1fhg, 1lc5, 1pmi, 1kt, 3hhy, 2ovc, 3imk, 3vn5, 1chd, 1g6a, 3qmd, 3mpk, 2tgi, 1ctt, 4ev1, 2v2, 3db7, 2xod, 2fyg, 1cvr, 3sgw, 3syx, 2atz, 2qfl, 4ga2, 2gjp, 3vgf, 3ey6, 2xij, 1tkj, 2yeo, 3df8, 2cfq, 1b1x, 4gos, 1wub, 3rlk, 1i5, 3odg, 3mzf, 3kw6, 3k1t, 1mw, 2mcm, 3en8, 1cfr, 3vgl, 3q7e, 2p8v, 2fk8, 3e7w, 4hd5, 3skp, 1u3d, 3kz7, 1b9h, 4jsr, 1gr3, 1ig8, 1hw7, 1znd, 3cui, 4mt2, 3sm3, 2o04, 2rbk, 2q8, 2hh6, 2r18, 3b79, 3u2, 4jx9, 2qjl, 4hbu, 3umh, 2cwr, 2p14, 3mcr, 3tef, 1qhx, 3tr6, 4kil, 1k12, 1sbp, 3pey, 1zcm, 2ict, 1ka1, 3afh, 4hc6, 4fe3, 1sz7, 1nrf, 1bc8, 1wwb, 3rj, 1xvy, 1bkb, 1he7, 3mcx, 1jil, 3zbv, 2xmz, 1wy9, 1ptf, 2wco, 4eo1, 3k89, 3kv9, 1nty, 3bk2, 1vk6, 1gdv, 1tvg, 1kwf, 2f15, 1ls6, 3a4g, 1hjq, 2fcr, 1wcv, 2wm3, 4i9o, 2hrg, 3co1, 3q8j, 3bqx, 1z6n, 3m0u, 3g0m, 2zic, 3tjl, 3klk, 2zwu, 2eif, 1xau, 1p9g, 3pua, 1p90, 1nd7, 2vfk, 4fgm, 3onk, 1g5z, 2b0v, 3noj, 3h04, 3a9g, 3h93, 4hti, 3fpi, 1lri, 1fp2, 1lj5, 2xrh, 1gqe, 1kuf, 1joi, 1p4c, 3rq0, 1czn, 2hzc, 2r31, 2d4p, 3ngk, 4au1,

3cqv, 1wna, 2yfr, 1q8h, 3ak, 1zma, 3ldc, 1x0t, 3h9c, 3sgg, 1oxx, 2ior, 1uek, 1orn, 3lcc, 1yz7, 2d7i, 2es0, 1mk0, 2gu3, 1qpc, 4iy, 2x7b, 1o6b, 1n26, 3st7, 4hl, 3vg7, 1pvv, 3tew, 1ew0, 4gak, 1kw4, 2o0q, 2i5v, 3ltj, 1e5k, 3h4y, 2x25, 1vbw, 4apy, 2opc, 2iu1, 3rzn, 3gbw, 2qru, 1j27, 3rju, 3n8i, 1cpy, 1n9b, 1wl4, 3q64, 1gz2, 1qdd, 4a41, 3qns, 1opc, 1u5p, 3s9x, 4avm, 1mxi, 1ds1, 2o0, 1t4w, 1n35, 3lyw, 2zzj, 1dt4, 3iqt, 2z4t, 1ej0, 3pfm, 3rio, 2ivn, 3bvx, 2rgy, 3hol, 3lrk, 3oa4, 2vq9, 3i8o, 4glq, 1nkd, 1bg2, 2d1v, 1pot, 2qc5, 2cbz, 2vg, 1rhs, 2oz, 3f7c, 1nx, 4fbz, 3l5a, 3k6d, 3gg7, 3m9z, 3bj9, 3ka8, 1ute, 2zhn, 4ggc, 1icx, 3k9i, 4bdx, 4ba6, 1rds, 2dyi, 3myd, 2wv3, 1ocs, 3pls, 2cyy, 1sjw, 2czr, 4do, 4fvq, 1o6, 3cm3, 3kpx, 4a29, 3mz0, 3i0p, 2jfr, 1u14, 1qh, 2x97, 3t8j, 2o4a, 3vnx, 2plt, 2p8, 2a66, 1ig5, 1uuq, 2fue, 3n71, 1fx4, 3ejf, 3f47, 2bbe, 3uw, 1ijt, 1dvo, 1ly2, 1rju, 3fay, 3o48, 3ipz, 3qvn, 1g6x, 3p7i, 1ac5, 1a8q, 3ils, 3ehg, 1iqq, 2ibj, 2hi2, 3c9, 3g9y, 1iwd, 2pqc, 2aaa, 3f85, 3gnp, 2nqw, 4iuw, 2ehs, 3cni, 1jgs, 3g8h, 3ksp, 3ii7, 1v70, 3osd, 3rc1, 3u62, 3bc9, 1j7g, 3d79, 2r8n, 1lp3, 2etb, 3iag, 3c02, 1cxy, 3a2q, 1mhn, 2vwr, 2pwq, 4jwx, 3rgo, 2ycd, 3o6, 3k1h, 3ftd, 3tg2, 2xom, 1c7k, 3zf, 3mqd, 3h9w, 2gkg, 1sjx, 1rmg, 3h79, 2cmt, 2a3n, 1hpt, 1mj0, 3vkw, 2zoo, 3aba, 1qd0, 2jgp, 3t95, 3tsh, 3o3y, 2ozf, 3thi, 1u7g, 1i7, 4hxc, 1s2x, 3lig, 3o4, 4gz, 1vd6, 3b4x, 3a9b, 2cu9, 4kf9, 1u84, 2hk5, 4e2x, 1gvc, 1q1u, 3hmc, 1bqc, 2jk1, 3dso, 1mvh, 1krq, 2qck, 1go, 3my2, 1j7x, 3c9z, 1sgl, 1txl, 2xl4, 2jk, 1guu, 2czl, 3e9u, 3hgb, 3deo, 4asm, 2via, 1mid, 1mqo, 2rjn, 1pie, 3cu4, 2p09, 3k93, 2ej9, 2wb3, 3alf, 3fia, 1cc8, 4hor, 3o12, 3g6s, 3ixl, 4hcj, 2o2x, 3kwz, 3a32, 2fph, 2w9q, 1xd6, 2p2e, 4euo, 3kwp, 2i4a, 3loi, 1vl, 1wzz, 2dri, 4j4w, 3qec, 3onj, 3vtf, 2y7b, 1zdy, 3icj, 2o7t, 1ruw, 3dm8, 3auf, 3kci, 3raz, 2wfo, 2wnx, 4dh4, 3ik0, 2qvg, 2rij, 1h6u, 1byi, 3t4r, 1lo7, 3f7x, 1b9w, 1wou, 3vi6, 2coq, 2o6n, 3r3q, 2w1r, 2q35, 3h36, 1lns, 1u3, 2p6w, 1a58, 1l2p, 1c7q, 4b6w, 1r29, 2i1u, 3s99, 2yvy, 3bw6, 2a9i, 2qhs, 1lyv, 3k66, 1m8u, 1wvr, 2hyj, 3a10, 4gnr, 3hdc, 3g1c, 3b21, 2a6z, 3snk, 2a8e, 2z2i, 1oll, 3rj2, 3lf9, 1p1m, 2wf, 3ggd, 1ki0, 1ek0, 3dnp, 3pmd, 1k8u, 2pln, 3ohs, 3ahc, 3iis, 3d2n, 2fqx, 1sat, 1ydl, 1zg3, 4jg2, 2i6j, 1vp8, 1ucs, 2b3h, 4h7w, 3pyf, 4hwx, 4f42, 3pzg, 1is1, 2obd, 1zir, 3b9w, 3klj, 1ar, 3qqa, 3vmm, 4htg, 1plq, 1zej, 1ws0, 2ywd, 2fgo, 3kxr, 1cwv, 1xk5, 3c3m, 3ian, 3hjz, 2plz, 1yo8, 4ht4, 1vjw, 4gpr, 3e02, 3pmo, 1jvw, 1xbi, 3bqw, 2pny, 3lmc, 1rwz, 1mun, 4h59, 2a98, 1ve0, 3g6i, 2o90, 2rff, 4g75, 2yd1, 8a, 2i1o, 2y2z, 2he4, 2xov, 1pch, 3my, 2ciw, 1lpy, 1iw, 3dfr, 3ewk, 1ciy, 3ejv, 2gqf, 1nnh, 2gui, 1gbs, 1m8r, 2xen, 3ht5, 1in, 4dth, 1dxy, 3rf2, 3h1g, 1p9b, 2vxy, 2vxz, 1g8a, 3sxs, 2gwm, 2e4l, 2z62, 2q4n, 1emy, 2ekl, 1u4g, 3clm, 3q2e, 4ici, 3kh5, 2oh5, 1qfg, 1vyf, 3io0, 3bjn, 1hg8, 3awm, 2g7o, 1izm, 3efa, 1c52, 1yfq, 3rnv, 3cdu, 2ciu, 1wq8, 2yxm, 3no2, 2ewh, 3mx1, 1z3, 1ozn, 2vbu, 1g2r, 3sc0, 3jwp, 4kor, 3gt2, 1ffy, 1dy2, 2ywx, 1mdl, 2fby, 2yci, 1xku, 2hng, 1xwy, 1m1s, 2yd4, 2of3, 1sn, 2duy, 3cyv, 2h36, 1nq6, 3fdb, 3iv3, 2o71, 3o4p, 1j77, 1tuh, 2hy7, 1fm, 2xj, 3oni, 3pqs, 1woz, 1vko, 1wnh, 3qgv, 3ir, 2ce2, 3o0f, 2osx, 2e4t, 1gk7, 1u2h, 1ywq, 3p96, 1i2k, 1oks, 1o73, 3o, 1xdn, 3jtm, 1e58, 1v9f, 2p4q, 3fci, 3voc, 2pjp, 2xbg, 3qky, 3eaf, 1sau, 3iar, 1zcj, 3ulh, 1zd8, 2jkb, 1h6h, 2vez, 2hew, 1wwi, 3lhl, 2qtd, 1zu0, 2ebn, 1ogq, 2uwf, 3f6f, 4jiu, 3sf6, 1hyp, 3ks3, 3hur, 1r1h, 1dp7, 4du, 1tuw, 2y78, 2vu5, 3qpd, 3bn6, 3lwx, 1f4, 1bxo, 3bs3, 1v3w, 1fob, 3nds, 1hh8, 3n, 2wzf, 1rh4, 2nls, 3sj6, 3tqh, 2zb3, 4exr, 1qys, 3l39, 2xvy, 1pdn, 1b1c, 3tjt, 3gd8, 1iv8, 2gn, 1zjr, 3td9, 2pjd, 4hik, 1ljo, 3v68, 1k04, 2bf6, 3zx7, 2zq7, 3pnn, 3n91, 4f0c, 2yzu, 1frd, 2ae0, 1wvf, 2a0m, 1v30, 2dkk, 1zgk, 1msk, 1o1x, 3md7, 3e3u, 1p5d, 4ed9, 2obv, 1h72, 2wge, 3dff, 1god, 2x5o, 1aly, 1g0c, 3dcn, 3luy, 2ggc, 2ygo, 3b7c, 2iiz, 1cyg, 2gzq, 1quu, 3pc3, 3bz6, 1uku, 1yh2, 3ach, 3cs1, 2xnq, 1c3p, 2hx0, 3txy, 2xc2, 2esb, 1zxq, 1xwi, 1tp6, 4gwi, 3uif, 2vm9, 3hr, 1m7, 3szy, 3sbm, 2ccv, 3ui4, 1y4w, 2vqc, 2pfz, 2zi, 1qhw, 1atg, 1pdo, 1uwm, 3slt, 3k1, 1mc2, 4eu, 2qa0, 3rjp, 1sbf, 3isq, 3ewr, 1aw, 2wfq, 3prn, 2g64, 1w4s, 3p0k, 3bpt, 1b0, 3ib5, 3ctr, 3ewn, 4g10, 2gko, 1qqf, 1vr3, 1b1u, 4ayo, 1fgy, 2a7, 1oc7, 4k9r, 3crv, 3i4g, 3vgi, 1yt8, 2q3t, 3dnz, 3c0c, 3n79, 3ph2, 2iwx, 3c1r, 1xw3, 3lv9, 1llp, 1od3, 1o0x, 3lmo, 1hx0, 3orj, 1edn, 1io1, 1wn2, 1npi, 2wp7, 3pe6, 1ea5, 2b1y, 3hgl, 1pdz, 2p3x, 3nrw, 3cec, 3fpw, 2nms, 1z6m, 2rhe, 1fbl, 3pm2, 1m6e, 1jov, 2az, 1vkk, 1s3i, 1fus, 4gb7, 3dt5, 2wf7, 1o4r, 1sn4, 1cza, 1r55, 1mvq, 3zn4, 4gmq, 2a11, 3kk8, 4ftf, 4gfq, 3g7, 1nn7, 2igd, 1gwe, 2nuh, 1bqb, 3cp0, 3p6, 3bwh, 4kds, 2ch, 2ar5, 4ano, 1qf5, 3f2e, 4gpa, 2aoz, 2xsk, 2evb, 3rqt, 2zue, 1u5h, 1moq, 1dlc, 3tpk, 3bjv, 1jyh, 4kqc, 1ccr, 1q2y, 3c12, 3puc, 3eki, 3ebr, 2r99, 4aeq, 3nv7, 3tvk, 3of1, 3ksr, 2gs8, 2okt, 1df7, 1w53, 2ywz, 2xgv, 1ah4, 3nmr, 2ovg, 2aq5, 3fim, 1b0y, 1qzm, 1sum, 1ni5, 3a0s, 4er9, 1sk7, 2xkr, 3a0e, 2nvo, 2pcq, 3vpy, 2ov0, 6ldh, 1c4x, 1kte, 2iim, 2nwf, 1w5, 3h38, 1wad, 1nls, 1hdo, 3foj, 3dkm, 3c70, 1p4p, 2p25, 2bfw, 1v6y, 3hx3, 1wa, 3mem, 3axs, 2bq8, 1gxu, 1j9a, 1p2f, 3pe, 1jj7, 3bkh, 2cc6, 3k01, 1tjy, 2hn1, 1vlc, 2w39, 2pk8, 1sen, 3cp5, 2y1y, 1w66, 1bgp, 3lrp, 2jdf, 2z6v, 1oa4, 1rc9, 3evt, 4b29, 3thg, 1xvx, 2qfe,

2v8i, 3k3v, 3boj, 3ln3, 1cnv, 1cfb, 4kk, 3av3, 3psh, 1z0f, 3e5d, 1gci, 1wlu, 3qa9, 3p7k, 3hnx, 3cm0, 1m9z, 2ch9, 2pst, 2v4v, 3b02, 1h16, 3l7w, 1i3u, 3f1j, 4gso, 3ggl, 3r6, 2azp, 3b33, 3vj9, 2bka, 2e3b, 1jfb, 2ccq, 1jl1, 3tys, 3o6c, 3a7r, 1c75, 1mvo, 1ccd, 1q2l, 1mjn, 2dh2, 1krn, 1ei5, 2sas, 2ics, 2z6o, 1fc6, 3fhf, 4dol, 3t2c, 2hgs, 3c0f, 1eh1, 4im7, 4kh8, 3id1, 1ji0, 1ls9, 1vcc, 4f1v, 1sv, 3hbe, 2wtg, 2eql, 3k9w, 1jf3, 3e8x, 3nh6, 1jf0, 1xdw, 3nqx, 1xt5, 1po5, 1mhx, 1pqd, 1bu3, 1cew, 2zpu, 3nbm, 1vqz, 2eyi, 1f1f, 3tbd, 3vwc, 1wd3, 3e21, 2vim, 1zy9, 2w15, 1t8h, 2x1i, 4ecf, 4eq9, 1dcq, 3mqz, 2hkd, 2rj2, 3iuu, 1nk, 1fo, 3nl9, 1x1n, 2fgs, 2g7, 1a34, 2auj, 1zy, 1bx7, 2j7j, 4f0r, 1fnd, 2cw8, 2z5y, 4iej, 3ek3, 2oyz, 2p4g, 3ira, 3cfz, 3c4h, 3gw2, 2ew1, 1klx, 2x7, 2xvs, 3bv4, 1c25, 3d02, 3rjv, 1pp7, 1nc5, 1wcw, 1fn, 1j5u, 1i5g, 3ayr, 4fd5, 2zzr, 2qg1, 3ifu, 1hcz, 2xsu, 2qia, 2ffm, 2yhs, 1kxu, 1x54, 2afr, 2nsf, 4glj, 1bif, 3bwx, 1i2a, 2rbc, 2fma, 4dza, 3akb, 2hxv, 3i4p, 3kxu, 3kvt, 2fe5, 2xeu, 1qb7, 3u83, 2jaf, 1eok, 2pvb, 2hkj, 3dgt, 2cn, 2j8g, 3e23, 2gv0, 2ccw, 3ap9, 2aif, 1o9g, 1eyh, 4dn, 4hc9, 1is, 2i2c, 2c81, 3qsq, 1tig, 1izd, 2o5v, 1jvb, 3f3q, 1cgt, 3ttc, 1j8u, 1z91, 4g2a, 3rsn, 3u4k, 3op7, 4hcu, 3ktn, 3f40, 2erw, 2cbp, 3gg, 2q8p, 4ev, 1k4i, 1md6, 4ksi, 4es, 1j6o, 2gbz, 1ofl, 2xz2, 2gli, 8dfr, 3fz9, 1yk4, 2r60, 4dzh, 2mhr, 2h9u, 1oal, 3riq, 1q9, 4hz4, 3e13, 2xjp, 2y9f, 4enf, 1k4t, 2jek, 3pf0, 1wdp, 1uly, 3ln9, 3ef6, 3hr8, 4jn7, 4j7, 3b55, 1xe1, 1wm3, 1buo, 3a57, 1iqz, 3gzi, 2p41, 1gmx, 2b4z, 2a0, 1knm, 1bkr, 1u2, 3e8y, 4edg, 1y79, 1noa, 1nm8, 3rnj, 2iw0, 2p4o, 1gs5, 3cjj, 1rzl, 3lq, 4gc0, 1htj, 1lkd, 2obi, 2i61, 3uq8, 4ipm, 2d4x, 3gmf, 1k7i, 2ar1, 1hdk, 3mj0, 3ocu, 2gnp, 1sr8, 2b0t, 1jeo, 3l5l, 1bhp, 1eia, 1f6w, 1esr, 1qhq, 3alj, 3nea, 1vb3, 1jjf, 3hz8, 1y6x, 3dlc, 3moy, 1tc3, 3cu3, 1isp, 2b0c, 4gk9, 4g1v, 1wdy, 1g9o, 3onp, 4fmk, 1o20, 3cu9, 3ctk, 3tz6, 2xrb, 3bz5, 1vip, 3dk9, 3fk8, 3g6m, 1ble, 2xs, 2v0u, 1ckt, 3emz, 3gr5, 3qkg, 1iuh, 3vco, 1xmk, 1hxi, 1aua, 2xpw, 3ny4, 3mvu, 1vls, 2eey, 4i3m, 1w4x, 2y39, 3q1x, 1k9v, 1upq, 2cg7, 3vk6, 3tt9, 3dsm, 3dz1, 1rv9, 3ohr, 3mng, 3i35, 1rr7, 3nzm, 2gy5, 3atv, 2gpr, 3trd, 1zpw, 1f7s, 3m3, 3uv9, 2ivy, 1szn, 2wdc, 3pcv, 4epc, 4jb3, 3kap, 2wao, 4esq, 2yxn, 2ie7, 2cyg, 4alg, 3l0a, 3f3z, 3ga4, 1dus, 2b4l, 1f7l, 3dg6, 3awk, 3vpz, 3w2z, 4ac1, 2c2q, 1l3, 3pka, 3i9w, 3qmx, 3evf, 3ie7, 3kb9, 1o22, 3d6j, 2pgf, 3a2b, 1rcq, 2zn, 1nzn, 1pmh, 3dx5, 3ss7, 2vfr, 3gk5, 1j2l, 4dnj, 1qoy, 3c97, 4ha4, 1m40, 1b6g, 4hvl, 3o8m, 1g3q, 3lzw, 2j07, 1y0m, 4a6q, 1pv5, 2bm3, 2z1a, 1o1z, 2fuj, 2h09, 1csn, 2d58, 2jku, 3lua, 3ojg, 2jcq, 3qvp, 1wta, 1dk8, 3uaw, 1ghp, 2ddx, 1z9l, 3i94, 1yiq, 1efd, 3c8u, 1fl0, 1mzv, 1q1f, 4b9, 3mxt, 3l6g, 2vs, 2rhf, 1c8c, 3jsr, 2f6e, 4j4r, 3lw6, 1ccz, 1p99, 2f9f, 2nw, 1uoy, 3mcq, 4dxy, 3d30, 2xdb, 4e12, 1owl, 4jhy, 4d8, 4e0g, 1ggz, 2hda, 3qc7, 1eaz, 1r26, 1drw, 1xp3, 2qyw, 3cih, 1z15, 2fz4, 3ziz, 3k1z, 1czt, 3pxl, 1t8k, 1ddw, 2o6q, 3e4g, 3iu5, 1nps, 3rga, 3qsj, 3mzz, 3zs, 2zpo, 1p9a, 2qng, 1oj8, 1b8p, 3ot6, 4e4r, 3by5, 3chm, 1omy, 1hlm, 2hxs, 1b4p, 2o9p, 3efg, 2aeu, 2ptz, 3fni, 1amm, 4e6s, 1arb, 2e9l, 2chr, 1yii, 3akh, 3apa, 3k7i, 2hd9, 2hps, 2c7p, 4f54, 2e8e, 1nhs, 1l9l, 1wvn, 1ulr, 1led, 3oo8, 2p6v, 3ufc, 1ps9, 2ejx, 3gm3, 2ywr, 3qz6, 3bzc, 1wba, 3q3u, 2pvq, 1pmt, 1qqs, 3isy, 2fg5, 3omb, 3ajz, 1up9, 2ra1, 3c1o, 2a4d, 4j94, 1mrj, 3g8y, 3i7a, 1spv, 3ost, 3eer, 1yzv, 2vpk, 1fzq, 1s35, 1h4a, 2vqd, 3ucx, 2xy1, 4hr3, 2fr2, 2wds, 2vpt, 1ft5, 3s2j, 3tfm, 2xki, 1dxh, 2imf, 2ggo, 3b4w, 3kwk, 2oit, 1vjk, 3fhg, 2vfo, 2j22, 3ml3, 3bwy, 1dfx, 4fbr, 2he7, 3kl, 2gwd, 1bq, 2bju, 1vyi, 2ftp, 3ka7, 1ht6, 1exr, 4kq9, 2q88, 3n7x, 2qjx, 2fdn, 1xcl, 1vpl, 2vb1, 3epr, 1qau, 3f, 1ops, 1qql, 2es9, 1w6g, 2ew0, 2rik, 3sd2, 1jer, 3t3l, 2qsv, 1n67, 2sn3, 1ulz, 2hvm, 2hkv, 1ri5, 3kyz, 3g2, 1dxs, 1z2n, 3tu, 1jix, 3cgm, 2qqy, 3eun, 1oyw, 2asr, 2o9s, 3scy, 2wbs, 2ja4, 1f00, 3jtz, 4gt6, 1s2o, 1muw, 3dmg, 1ubd, 2fuf, 1t6c, 2p0b, 2wi8, 3cg3, 1lvl, 1w99, 1u3e, 2pjz, 4a1k, 3r20, 3hlf, 4ex8, 1uby, 3gbe, 2dq7, 2gno, 1w4t, 1i6p, 2dsx, 2yik, 3mwc, 1wch, 4f2f, 1nf, 1m53, 3ewy, 3o3x, 3t0h, 1w3w, 1zzm, 3hh8, 1vfy, 1td4, 1nrw, 4fxc, 2e2c, 2boo, 3mdq, 3lmz, 3kya, 4dmv, 2ge, 2vkl, 3oz2, 1rw1, 3rfn, 3klb, 1xak, 2xu2, 1h70, 3a0f, 4i5u, 3ty, 4hu2, 1os6, 1oxj, 1hzt, 1uwv, 3m1x, 2aj6, 1e0c, 3dqp, 3hmz, 3s64, 1zbm, 2bv5, 1ib2, 3exq, 2fbh, 2i06, 3t7a, 1ua4, 3pl8, 3idv, 2z99, 1xkn, 2vac, 2es, 3nyy, 2a0j, 3rt2, 3lu, 2gs5, 2hc8, 1umk, 1o5l, 3ory, 3l4r, 2wq1, 2ayd, 1sqw, 1a7g, 1thx, 3eur, 3zu, 4b2f, 2fmp, 1vmg, 2c2u, 1h0p, 1gen, 2pmp, 1gyc, 1zo, 1q92, 1bhe, 2zj8, 2fm, 2qyz, 3c7a, 1uxo, 2xfd, 1dpe, 3a9, 1z7w, 1rcd, 2h2, 2gk, 1wv8, 4hbz, 3pxh, 4jb7, 2i53, 1coi, 2fgc, 1rmr, 4ehx, 3h5q, 3ioh, 1to3, 3r0x, 4hv, 1x8q, 1ybh, 2yl6, 2pkt, 3ijf, 1w0n, 1czj, 3g1t, 2nwv, 1bb9, 1rtq, 3p5u, 1qr, 2pi6, 1ys9, 1yt3, 2bjf, 3t7l, 1ru3, 2oj4, 2gsd, 2uv4, 1gpr, 3kyq, 1bh6, 3a8u, 1uj8, 2qc, 3dee, 2zeq, 2ocg, 1uxy, 3r5t, 3h6q, 1mpu, 3rjs, 2ftb, 2a15, 1zin, 1uai, 1yw5, 1cby, 2zpt, 3gy9, 3qm, 3aap, 2vd2, 3lhs, 3b8a, 1jc9, 3kao, 3ff4, 1v0d, 3pdx, 1rtt, 2bjq, 1n5, 1xs5, 2c71, 3le2, 3tn2, 3rx6, 1y, 3pyw, 1poc, 1lg, 3bmv, 2ykz, 3q8i, 1qw2, 2vbk, 3ceb, 2ahn, 3fdj, 2hxp, 3nsz, 3oc8, 1msc, 1lbu, 3d3y, 3gce, 2z8x, 1ef, 3rmi, 1lxi, 1u2k, 1wu, 3q2, 3vny, 3cla, 1oz6, 1r5l, 1ucd, 1ed1, 1use, 2qsb, 1rm8, 2cih, 2apl, 2haf, 2gke, 1u9c, 2uvj, 2hrb,

1qyi, 3k21, 1nar, 1zlb, 1m15, 3dgz, 1n9, 2bkw, 2jc4, 3vqf, 1m1q, 4ffk, 2oln, 1fhe, 2znd, 2hm7, 3swg, 1sdi, 3mb5, 3hfi, 3l92, 1d7o, 3exv, 1mw7, 1bik, 2r9f, 3lx, 3enu, 1iom, 3nh4, 3il6, 3pf2, 1r4x, 3p51, 3u5v, 3g87, 3qnm, 2cm2, 3irp, 4h08, 3q69, 1fg7, 2qml, 3rh, 3fvz, 1fi4, 2y1b, 1nuy, 3fxz, 2cw9, 2cal, 3iv4, 1z4r, 3r4t, 3fp5, 2y6h, 1yzm, 2ehg, 3hqx, 3ptw, 2e0t, 1yhf, 4ioh, 1f0n, 2ay, 1q9b, 3dv8, 3u64, 1c0p, 1dmu, 2pth, 2qk1, 3f04, 3oig, 1k0i, 1ukz, 3na6, 1t9f, 2erl, 2sli, 1vgp, 1uv4, 2qn0, 2abk, 3h7u, 1bh, 1ygt, 2xyb, 3d6l, 1zod, 3zm8, 2yxb, 2xn2, 1sur, 3pm, 3q1, 2oxn, 2g9d, 2h2b, 1edt, 2xcz, 1jmc, 3fpl, 1ulv, 3ka, 3uar, 1e6u, 3a7n, 3lhc, 2xb4, 2hbj, 3f7l, 1qf9, 3dvf, 1ne, 3ecm, 3s5q, 4agk, 1oot, 3zzo, 2ygt, 3a3j, 2pcn, 3hza, 1lmi, 3un6, 3civ, 3n0q, 1qtw, 5cy, 1aw9, 1fqi, 1mi, 4fs3, 3bxv, 3bwz, 2plc, 3qvs, 3slr, 3tpa, 3hd4, 1zvd, 1dlw, 1tu9, 1vjz, 1s7z, 3r72, 1eov, 1i5p, 3bwt, 3h92, 1i27, 3swe, 2r0x, 3tu8, 1cbg, 1hbk, 1vpe, 3bbl, 2i0x, 3pr9, 3t7z, 3hyn, 2haz, 2fsq, 2a4, 1bfg, 2xf, 1cjc, 1qnx, 4hka, 1mtz, 3iij, 3see, 2hh7, 2i9i, 2z84, 1a76, 4f8x, 2x3m, 4ham, 1mgt, 3zp5, 2o, 1x19, 1jhs, 4g63, 1vc2, 3ebh, 1vjo, 3gms, 2ebf, 4h8, 4f5u, 1paq, 4hno, 3r2g, 2onu, 1cpq, 3ups, 3to7, 2ek8, 3zv, 1ej2, 2b5w, 2woy, 3gru, 2cwy, 1xv5, 3feu, 2r75, 4aiw, 2ou6, 3re9, 1sfp, 3fg2, 1l1s, 3rhg, 1f2v, 1jhg, 1jb3, 1cyj, 1p2x, 3cg4, 1n7e, 1ub2, 2gxg, 3zyz, 1pgv, 3ksh, 4g9y, 3o1c, 3oj0, 1ojq, 3h6j, 1pz4, 2ixa, 1ttz, 1ei9, 2hcm, 4evf, 3t33, 1nkr, 2bgs, 2c4x, 2dwr, 4f0f, 2vpj, 3bjo, 2x9z, 2iay, 2ozp, 1xyi, 2o8n, 1g43, 2xdp, 1rra, 4he7, 2be, 4b21, 1vtn, 2p51, 1z6g, 2aiu, 2cxh, 3gbo, 2xj4, 2yyz, 3laf, 3lqn, 2cy5, 3mvs, 3djl, 1imj, 3gkj, 2xr, 1q25, 3jxy, 3t9w, 1g9g, 1dy, 2vsd, 3ck2, 1zch, 2d59, 4jcc, 3f3x, 1nrg, 2w3w, 4b4y, 3ee4, 1gu3, 3f7s, 1l6, 4dq, 1lzl, 2iw1, 4f85, 1lgp, 3ml5, 4ew7, 3vtg, 2p26, 1c9h, 2o14, 4e4u, 2of7, 2f2b, 1wmd, 1gh2, 4gl3, 4jwo, 4j2h, 2qqi, 3qwp, 3n4j, 3cjm, 4a4a, 1hlv, 2r2y, 2uxy, 1ni3, 3lwt, 1b0b, 1bud, 3d1p, 2cxc, 1io0, 1lvg, 2ei4, 2f5t, 1hz4, 3b34, 3tdw, 4er8, 2vgd, 4fyj, 2hs, 1ti, 1vrm, 2v5i, 1jx4, 1hyq, 1ux8, 1ywm, 2yvt, 2o8l, 4hpz, 2e1f, 2ovj, 3bb0, 4cpv, 1acf, 2rhw, 1bl0, 4hfv, 3v75, 1zv, 1vbg, 2ii2, 3bs2, 2e8, 2v4n, 2xx, 2x49, 1y8c, 2bv6, 1mkp, 3s44, 1q33, 2yih, 2gen, 3olc, 2ce0, 2toh, 2p7s, 1g5t, 3etp, 2o8p, 2w86, 3p3y, 1r8i, 1x3s, 2fb0, 1ml4, 1rfe, 1e4m, 2eh3, 4aa9, 3pb6, 3elx, 1gbg, 1pq7, 1fw1, 1qlm, 1skn, 1vk9, 3i8t, 1uy4, 3mdm, 3dih, 1hvx, 2gdz, 1vyr, 2e3, 1aho, 1urr, 1vlo, 2cy2, 2zpm, 3ce7, 3llx, 3dsh, 2eaq, 2poi, 1fxd, 2gtq, 2imq, 1ne8, 2ylm, 1bh0, 2d8n, 2bzg, 1vf8, 3tre, 2e2r, 1rwr, 4a4j, 3kwe, 3i8, 3i03, 3kq0, 1kao, 1xyn, 4fcu, 1j8e, 3a5v, 1reo, 3ql6, 3ckm, 3r5e, 2cy3, 2p65, 3po0, 2h8e, 4hq1, 4aio, 1wdn, 3qa, 1ee6, 1idk, 2yhx, 1ro2, 3sc7, 2au7, 3kiv, 1r89, 1at0, 2rb8, 1usg, 2pqx, 4k1o, 1ohl, 1uru, 3tow, 1z81, 3m6w, 3p1g, 1jwq, 2vri, 1upi, 7fd1, 1flj, 3peb, 1uha, 3v39, 2yvi, 3mwz, 3acx, 1mx3, 2rdq, 3goz, 3q1n, 2j8h, 2c4e, 3buj, 4k3f, 1k1a, 2xd3, 2i9c, 1l6p, 3ibh, 2hhg, 1cuo, 1j3e, 1w41, 1dur, 3hft, 2d5b, 1tt8, 2fq4, 2zvc, 3qex, 3zt9, 2dvz, 3o59, 4dg5, 2odl, 4hty, 3s69, 3s9j, 3rpw, 4hqu, 4ezi, 3q2u, 3m70, 1d5c, 1j24, 3cbg, 2g3r, 1lc0, 3vxj, 2d3y, 3ti, 1h12, 1tgj, 3oz, 1jf9, 1vd3, 3o2g, 4grz, 2i5u, 3skq, 4bh, 3oih, 3d80, 3p0f, 4gh9, 2e1z, 1kp6, 2o9g, 1kq6, 2oe, 3f5d, 2ywk, 1yrv, 1ux5, 2nr9, 1ud2, 2yyo, 3fzx, 3csk, 3oc9, 2ex2, 2evr, 4hqa, 1hu, 3q1h, 1pex, 1bea, 1dzf, 2xwp, 2wui, 2fd5, 1dxj, 2g7e, 3u97, 4fr9, 3igh, 2h1v, 1hpi, 3bja, 1j09, 1nj1, 3l5i, 3sul, 2chd, 1i52, 3p3c, 1apa, 1uds, 1g8s, 1doi, 1r69, 1hk8, 2w0g, 1ihk, 1yfu, 3mwf, 1iuz, 2g2d, 1uk8, 1frb, 1jye, 1fao, 3nmq, 2cdn, 4gz9, 3k6y, 2p8b, 3qxc, 4iil, 3qy3, 1hg7, 3hjr, 2bi, 3k7n, 2j4o, 3mva, 2wnp, 2g3a, 2cxy, 3c19, 1nwa, 2o4t, 2fb6, 2rk3, 3r26, 1ixl, 3r4c, 2i49, 1r18, 4h8q, 1rb9, 2zqe, 3uk0, 1n3y, 3ir4, 1o8v, 3oos, 2agc, 1ujc, 3s92, 2j8, 2esh, 2vf, 1j98, 3hvw, 1pyf, 3f5r, 3a1k, 2hoq, 1p3c, 3sh4, 2vhk, 3jtg, 2guy, 3edy, 1yal, 1mh8, 3bl6, 1fjj, 2y9u, 3go5, 4f7h, 3fbl, 3iix, 1xte, 3kn1, 3lk7, 3kdw, 3c7x, 1hnj, 3c5k, 2ze7, 3mao, 3s9f, 2pj, 1lko, 1omr, 4al0, 3qou, 1clc, 3tx2, 1t3b, 3hbf, 2gw, 3l8w, 1i39, 3r2p, 3ok, 2plh, 3vhj, 3ozz, 3exn, 1ear, 3o5q, 3hmr, 1jug, 2c3g, 2rk5, 1m85, 1mba, 3dnx, 1j5y, 2y24, 2fl4, 2gpj, 2ece, 2qzs, 4fey, 2q8w, 1wu3, 1vbl, 1o6d, 1xaw, 2gsj, 1ifc, 2noo, 2zib, 2d3n, 1n7o, 1kfw, 3qom, 1agx, 3cl5, 1twu, 3f2u, 1zyb, 3ig5, 3sk, 3l1e, 2wz9, 2w4j, 3qh4, 1mof, 1lxj, 2dp9, 2zw0, 2wfi, 1kdj, 1qm7, 1iiz, 3ay8, 2och, 1sgw, 3qao, 2hzh, 1jdl, 3gv3, 1rpa, 3p3o, 2h5u, 4f06, 1dhn, 3qc0, 3qwx, 2qyb, 4aoh, 4jzc, 3teg, 2yb6, 1ra0, 3gc2, 3zvl, 2ozg, 1es9, 1rkd, 2i6v, 2nlr, 2jlq, 4dt4, 1fx, 3qfu, 3lmf, 3nwg, 3c7f, 1jq5, 1sl8, 3u2a, 1cc5, 2dho, 2ob5, 1yo5, 3dgb, 1gh, 2qlu, 3ju4, 3sv0, 1nb9, 4hx, 2on8, 1uj6, 2fiw, 1r6, 3npd, 2yz8, 3zqu, 3u81, 3n7t, 4a3z, 2hq6, 2go2, 4exo, 1qaz, 2wno, 1scz, 1oe1, 3exm, 2r0j, 3mbd, 5nul, 2gwn, 4fd0, 3sy1, 3isx, 3vfi, 3w1, 3pfg, 4i6o, 3b49, 3vor, 3rrk, 2nx2, 2os3, 3m9l, 1khi, 2pn, 1m7b, 3qe1, 2wsl, 1hlb, 2xbt, 4huz, 3odp, 1ezw, 4asl, 2oze, 1nio, 2dbq, 2oxk, 3adj, 3d0j, 2ppv, 3hc1, 3l84, 2cfe, 2bk8, 3kqn, 1g66, 1p9, 3trk, 4j9, 4esm, 4e4c, 3kkq, 3tbn, 2pl1, 2b71, 1vk2, 1goj, 2i88, 4a35, 3hvv, 1qwz, 2e7z, 1izc, 3vl, 2fi0, 4atm, 3ziy, 3ra, 1mc0, 3b0t, 3rmq, 3i5a, 1d4o, 3vog, 3ip0, 1uiy, 3m3g, 2hyv, 3ksx, 1xr5, 2ja2, 1awd, 2y7p, 2ozh, 3rfy, 2b69, 3zh, 1zbp, 1efy, 2fvy, 1ydx, 2cc1, 3hrp, 3ey5, 1qcz, 1aol, 1sny, 1oh4, 1znw,

1paz, 3ein, 1bgf, 2yby, 2f1n, 1k7c, 3kfw, 4euw, 4fvg, 1mdb, 3tr7, 3f14, 3tgl, 3c9p, 3fo8, 1r03, 3l22, 1jae, 2ww8, 1t3, 1uok, 2fn4, 2vu9, 1zp2, 1i6l, 4e9a, 2b5e, 3plw, 3n37, 2ea9, 3rdr, 1oht, 1c1k, 1f2j, 3zsj, 2ztg, 3nf2, 3f3, 4dx, 1zbs, 2pm1, 2z51, 1yqe, 2wsw, 3due, 1r2q, 2xw, 1v0l, 3hrg, 2bmw, 4eih, 1td6, 1ew4, 2ejc, 1jv, 2ze3, 4gc3, 1bd8, 1x46, 4j29, 2gs, 2cfm, 3aam, 3bgs, 1wm, 3n4h, 1nh1, 2x5x, 3rkv, 3l6n, 3m5q, 2p84, 3l2c, 1ej8, 4eo9, 1byp, 3b64, 2yfu, 4b28, 1i77, 2pe4, 1ka2, 2xmf, 3ic4, 3hj7, 1r85, 2asb, 3bod, 3gir, 2z10, 1whz, 3gia, 1vaj, 3vmv, 3osx, 1yd0, 3qh9, 1elj, 4gw, 1rm, 2oyp, 2qzq, 2v9v, 1ku3, 2cro, 2ya0, 1zjc, 3g4, 1wrj, 3f4s, 1nfp, 3ea6, 3m66, 2et1, 3iu6, 3cwi, 4gei, 2p0k, 3l2a, 2i2q, 2zc1, 2vec, 1khx, 3dn, 3js8, 1ojt, 2p8g, 4dzk, 2qrl, 2vc8, 3hz7, 3cao, 3n0x, 3zz, 2vq4, 1pbj, 3kyl, 2oeg, 2fz, 1eyl, 1esw, 3lp5, 1wka, 1fx2, 3smz, 3r0v, 3rx9, 3st1, 1thf, 4eiu, 2nap, 3cgh, 1phk, 1l2, 2v82, 1h8l, 1gp6, 1siq, 2end, 1tis, 1kwg, 3cnu, 3ib7, 2af0, 1yqg, 3ngp, 3msh, 2oo2, 1ouv, 1r6d, 2rae, 3kr9, 3gmv, 3igz, 1wde, 2qcp, 1fvg, 1lrv, 1vff, 2hxt, 2qnk, 3zr8, 3b84, 1z8o, 1odh, 3poh, 1sqh, 3gq8, 1a8d, 2hai, 1kfn, 3tqz, 3os, 3ry7, 1pf5, 3fz4, 1sek, 1rgv, 3rh2, 3f4k, 3eau, 1ypb, 2op6, 1xr6, 2erf, 3oc6, 2jen, 3fd3, 3ros, 2psd, 2pk, 3m48, 1btn, 2p8r, 3uci, 3d3z, 2cu2, 3lup, 2o36, 1yzg, 2pkp, 1tzv, 1d3c, 2vxn, 3bch, 3jyn, 3ouq, 1h75, 3lez, 1jg1, 1a8l, 3pkv, 2q4u, 3sg0, 1oaa, 2vn4, 1r8n, 3iee, 2xfr, 3fh2, 3a7i, 1iow, 3o9p, 3ono, 3t57, 3aj7, 4e1s, 2zyo, 1dyq, 1tqg, 3erv, 4ecg, 1jsg, 1mf7, 1qho, 2v14, 1ise, 4a9v, 3qpa, 1c8z, 3elo, 3ftt, 1vkw, 1vb0, 3dcm, 2o0m, 4fei, 1jko, 2olr, 3ls0, 3us6, 3kt9, 3gt5, 1an8, 1ig7, 4jm1, 3r2k, 3sx6, 3hzu, 3sil, 3d2w, 2gsq, 2bo1, 2iwa, 2nvn, 1eca, 1nkg, 1o06, 3lf, 1gzc, 3vs, 4gdz, 2fj8, 1mug, 4ax1, 2g9, 1zt3, 1roc, 1csh, 3ba1, 1ex7, 2d48, 2j8k, 2cir, 1qam, 1s8i, 2gkp, 1jos, 3tx8, 3cq1, 1r5a, 2x8x, 1n83, 3dml, 2d3d, 1o1y, 1r1m, 3l4h, 1a59, 1olr, 2xqh, 1ihg, 1mix, 2y8b, 1nf9, 4eb0, 1s1p, 2cbn, 2x7q, 2yk4, 2v2p, 1iag, 1pjc, 2ip6, 2v72, 3iib, 3um, 3k62, 3r2, 1eje, 2haq, 1dun, 3gon, 2w9h, 1ex9, 1h98, 3klr, 3aot, 1p6p, 3k1d, 2am1, 2imp, 3ieh, 4idc, 1gwu, 2qnw, 2fzp, 1qkk, 2wy4, 3w06, 1a8, 1jid, 1zk4, 2as, 3ose, 1ocy, 2fc3, 3il3, 3cgx, 3pms, 1iat, 2v75, 1d2n, 3aj3, 2ydl, 3azc, 1ll, 3dkr, 4e0h, 1cot, 1pcs, 4bfg, 3dai, 1nsj, 1cf, 3le4, 1hnf, 1jf8, 3nw4, 3ljk, 1wst, 1s2m, 4jgl, 1pj5, 1em7, 1e5m, 3ag7, 3hzs, 3rab, 1bt0, 2fcl, 3oao, 3te4, 1r6x, 3ugo, 3shs, 3mk1, 3jyo, 1r4v, 1jak, 2fdr, 1pq, 2ojh, 2gdm, 2cx1, 2zj3, 1eb6, 1g7n, 2z17, 1tjx, 2hyz, 2pim, 1cb8, 3lho, 4acj, 3pmm, 2in0, 3q6o, 1t1d, 1t2d, 3lyg, 2xu9, 4go, 3trg, 3im1, 2e0q, 2wqd, 1xmt, 1rmd, 1tib, 3hoi, 2wh7, 1cxc, 2p52, 1lst, 1rqb, 3kl7, 1tn3, 2xr6, 4hs9, 2qsk, 3tx6, 1wqg, 2cvb, 3h5i, 2vk9, 1wrm, 3qnt, 3c0i, 1e4i, 2cnd, 3pp5, 1cnu, 1zhx, 3kjh, 1ctj, 3uv5, 2oqr, 1bm8, 3l9s, 2qc3, 3hvm, 3a02, 3lpc, 3to5, 2v1, 2yok, 2fq3, 3zyq, 1st8, 3bs8, 1id0, 3s8m, 4eq3, 2hdz, 1rl0, 4aq4, 1z2a, 3noh, 2hyt, 1p6, 4grf, 1hfx, 1vmi, 1kw3, 1ve1, 4dp1, 2act, 2d0s, 1zox, 1bdo, 1b56, 3oj7, 4h1x, 1q5z, 3jvl, 3nv1, 3a7l, 1zsq, 1gq8, 2a7b, 3sny, 3gzk, 2im, 2w5q, 1qz9, 3off, 2a0b, 3bfm, 1zm8, 2h30, 2eve, 3bcy, 1gqn, 3boe, 1t7v, 3hcz, 3qst, 2ixm, 3kh7, 1r6v, 1tyj, 1aun, 1xub, 3ii2, 2pww, 3gfo, 3fym, 4e6u, 1n5u, 2hyk, 2jfu, 3e8t, 1cyx, 1rl6, 1crl, 4ha6, 3iev, 1dz3, 3tdg, 1juv, 3es1, 1nhy, 3iwl, 3uaf, 3co6, 3dxt, 2fu0, 3mf6, 1mil, 1x38, 2r51, 1dhr, 2waa, 3by8, 2cjj, 1wd5, 1pa2, 3tr0, 1lba, 3zy, 1qnr, 3s6k, 1bf2, 3ed5, 1c3m, 2w51, 2obl, 1ycc, 3kkf, 1f20, 1dv8, 3fs, 2qk2, 2v5m, 3qok, 1a70, 2zb4, 1z3d, 3kyf, 3bg8, 3hut, 1i2t, 2prd, 1ep0, 3s1y, 4i6x, 2ns0, 4kvf, 2cwp, 2b49, 3so2, 1ae, 2f7f, 4dvc, 3n1u, 1rlk, 2gtg, 1kth, 3gb0, 1wri, 4f7a, 3qd7, 3okq, 1tuv, 4dzg, 4f8a, 3e7p, 2gvi, 1mzy, 1xww, 1kng, 2qff, 1edq, 2ywj, 3pwz, 1hus, 1cl8, 4eby, 1li4, 1wma, 2vo8, 4fe9, 1agi, 1fxl, 2xbf, 1k6k, 2e8b, 3zsu, 1rro, 1v2x, 2j6a, 4a3, 2hah, 4i8i, 2dt8, 3vpq, 2e7u, 1iz, 2efv, 3rfw, 1l1q, 3snf, 2y3c, 4f98, 2ihr, 1ew3, 3i9x, 1bup, 2pa1, 2j0w, 2pt2, 1qqy, 1byr, 1lu1, 4a20, 1qst, 3cfy, 3h41, 1w2l, 4inw, 3css, 1jl5, 3mtw, 3nph, 3mf7, 3k29, 3l8d, 2p17, 1x9g, 2d81, 1tpy, 1o98, 3phs, 4ee9, 2q0z, 2y6x, 2jfn, 2vq, 1ug6, 1odm, 1dgj, 1tr, 1b74, 3zsc, 3aa, 3ljn, 2v3q, 1ktb, 3ibt, 3p3g, 3mtc, 1c3, 3dan, 3lx1, 1eu8, 2xxp, 3ng, 1xhd, 4gqr, 1nog, 1ut9, 1j58, 2xws, 3q2b, 1z24, 4hua, 2asf, 4a02, 1wm1, 1zr6, 1vjf, 3r9m, 1x91, 1vfl, 2hzq, 1c0a, 2jar, 1z3i, 2zhj, 3ar4, 3ni2, 3cs3, 1u6t, 3bln, 3no3, 3jyz, 3zui, 1qre, 1xeo, 1vg, 1ji, 3f2z, 2qr7, 4f0j, 2qlt, 4htl, 1nhl, 3di5, 2x5r, 3hnr, 1a6f, 1w8i, 2pvu, 3vm7, 1cqq, 3lxy, 3rja, 1o8x, 3po8, 3et, 2qpx, 1yhl, 2g5x, 1wy3, 1ok0, 2qmq, 4e9l, 1ch, 1f1s, 4ekj, 3eln, 1ij, 1uoh, 1t07, 1hsk, 1rwh, 1udc, 1q5, 1qhd, 3oou, 2o0b, 3dew, 1vi7, 1al3, 2q3, 1hup, 2fba, 1vly, 2qim, 4hz8, 2o3e, 2ze9, 2bh9, 2jdc, 3mb3, 3vm, 3f6v, 2ftw, 2bf9, 2qvt, 2o9, 2c8s, 3sz7, 2plq, 2nr7, 2r58, 3s79, 1i0v, 1hh2, 2hqj, 1mzp, 3ezo, 1lng, 1ks9, 3qqz, 2p02, 1af7, 1qwo, 1gs9, 3oiz, 2q9v, 3qap, 2xio, 3bkr, 1hh5, 1sk4, 1hq8, 1le, 2waw, 2z5w, 3cuz, 3lwd, 1gpi, 2ehz, 1m2k, 3i4, 3g21, 3rpz, 3im3, 1f3z, 1sif, 1z1e, 3ue2, 1tlq, 2pne, 3gjy, 2w2e, 3bh0, 3mjf, 3e8v, 3nz3, 1o88, 3e6j, 3fi7, 1xeu, 2gqt, 2xsp, 2rh3, 1iam, 1t10, 2ac1, 1cdw, 1c1l, 3d9h, 2z72, 1y93, 3k8u, 3a99, 2w91, 4f0w, 3rvc, 1gxq, 1d5t, 3qoo, 1dpb, 1y0p, 1bj7, 1yn4, 1ua7, 3ooi, 3gvo, 1f94, 3atr, 1ws6, 2y7, 2bvl, 3kb5, 2g7g, 2r0b, 1ogo, 1nth,

4e4y, 3hje, 2dxa, 2zcw, 1iej, 1s68, 2rci, 4bi6, 1inp, 3ioq, 2rfa, 3hro, 2oss, 3qdp, 1i6, 2hjs, 3kfo, 3l7n, 3bdi, 1kkh, 1vsr, 3qs, 1gvp, 3ah7, 3s9k, 2jg6, 2z4s, 2bop, 2csg, 1ts9, 3uhm, 1ln4, 2r2z, 1rc, 2fnc, 4ipi, 3pvh, 1zce, 3ghf, 1jhj, 1pb1, 1j4n, 2y3z, 3s0a, 1euw, 3m7k, 3lwj, 2c9a, 1ff4, 4i1l, 3zx1, 2wiy, 1cyo, 4ato, 2b9w, 1thm, 1fk5, 3ax, 3idw, 2aan, 3g9h, 1kt6, 3q3q, 3r5l, 3skx, 1ks5, 4fk, 2vyw, 4eo0, 3qy7, 3ng7, 2qip, 2au3, 2fgg, 3ii1, 1z7h, 3sng, 16pk, 1ci3, 1civ, 2pmk, 3tch, 3llv, 1seg, 3n90, 2edm, 2o3g, 2vuv, 1thg, 3zta, 3nzb, 3dxy, 1aba, 2ppx, 3ncl, 2hw2, 3b09, 1t1, 3quw, 2zgc, 3kxt, 2zk9, 1rlw, 3vc0, 1xgw, 5pal, 1axn, 1nij, 3dq, 4hhf, 2o4, 1zlm, 4g08, 4pfk, 1t2, 1psz, 3das, 1aie, 1ion, 3onh, 1ri6, 3jsz, 1ryo, 2xbk, 1yoa, 3r6k, 4aiv, 1n9l, 1jr9, 3nzl, 2r0, 1oz2, 1qwg, 1lj8, 2d0b, 2hs5, 2vx5, 3v46, 1ewf, 4hd1, 2bep, 1ui0, 3ld1, 1zua, 3cb6, 2q0y, 2zxy, 2olg, 1h45, 3b2n, 2fi9, 3h7i, 3zri, 2z61, 1ja9, 2ici, 4km5, 3voa, 2hc9, 3i0w, 3l78, 1iiu, 1ifr, 2w8n, 1h7c, 2ify, 3o9, 3gf8, 1j0, 1e3j, 2yo2, 3ers, 3ouv, 2cjg, 2atm, 3dxs, 3c0w, 1t95, 2z0m, 2iih, 1dd5, 2huh, 3gg6, 2pii, 1q0r, 2ia7, 2qi, 1ng2, 3va9, 3uwc, 2nsc, 1c7s, 3r2q, 1mgr, 3soy, 4gi, 3u9w, 3ado, 1vhe, 2hx, 1n5d, 1kn3, 3pdg, 3ht1, 2sfa
